# Heterogeneity in the diagnosis and prognosis of ischemic stroke subtypes: 9-year follow-up of 22,000 cases in Chinese adults

**DOI:** 10.1177/17474930231162265

**Published:** 2023-03-16

**Authors:** Matthew Chun, Haiqiang Qin, Iain Turnbull, Sam Sansome, Simon Gilbert, Alex Hacker, Neil Wright, Tingting Zhu, David Clifton, Derrick Bennett, Yu Guo, Pei Pei, Jun Lv, Canqing Yu, Ling Yang, Liming Li, Yan Lu, Zhengming Chen, Benjamin J Cairns, Yiping Chen, Robert Clarke

**Affiliations:** 1 Clinical Trial Service Unit and Epidemiological Studies, Nuffield Department of Population Health and Big Data Institute, University of Oxford, Oxford, UK; 2 Department of Engineering Science, University of Oxford, Oxford, UK; 3 Department of Neurology, Beijing Tiantan Hospital, Capital Medical University, Beijing, China; 4 Oxford-Suzhou Centre for Advanced Research, Suzhou, China; 5 Fuwai Hospital, Chinese Academy of Medical Sciences, Beijing, China; 6 Peking University Center for Public Health and Epidemic Preparedness and Response, Beijing, China; 7 Department of Epidemiology and Biostatistics, School of Public Health, Peking University Health Sciences Center, Beijing, China; 8 NCDs Prevention and Control Department, Suzhou CDC, Suzhou, China; 9 Medical Research Council Population Health Research Unit, University of Oxford, Oxford, UK; * Contributed equally; † Jointly supervised the work; ‡ Members of the China Kadoorie Biobank Collaborative Group are provided in the OnlineSupplement

**Keywords:** Ischemic stroke, etiology, classification, machine learning, China

## Abstract

**Background:**

Reliable classification of ischemic stroke (IS) etiological subtypes is required in research and clinical practice, but the predictive properties of these subtypes in population studies with incomplete investigations are poorly understood.

**Aims:**

To compare the prognosis of etiologically classified IS subtypes and use machine learning (ML) to classify incompletely investigated IS cases.

**Methods:**

In a 9-year follow-up of a prospective study of 512,726 Chinese adults, 22,216 incident IS cases, confirmed by clinical adjudication of medical records, were assigned subtypes using a modified Causative Classification System for Ischemic Stroke (CCS) (large artery atherosclerosis (LAA), small artery occlusion (SAO), cardioaortic embolism (CE), or undetermined etiology) and classified by CCS as “evident,” “probable,” or “possible” IS cases. For incompletely investigated IS cases where CCS yielded an undetermined etiology, an ML model was developed to predict IS subtypes from baseline risk factors and screening for cardioaortic sources of embolism. The 5-year risks of subsequent stroke and all-cause mortality (measured using cumulative incidence functions and 1 minus Kaplan–Meier estimates, respectively) for the ML-predicted IS subtypes were compared with etiologically classified IS subtypes.

**Results:**

Among 7443 IS subtypes with evident or probable etiology, 66% had SAO, 32% had LAA, and 2% had CE, but proportions of SAO-to-LAA cases varied by regions in China. CE had the highest rates of subsequent stroke and mortality (43.5% and 40.7%), followed by LAA (43.2% and 17.4%) and SAO (38.1% and 11.1%), respectively. ML provided classifications for cases with undetermined etiology and incomplete clinical data (24% of all IS cases; n = 5276), with area under the curves (AUC) of 0.99 (0.99–1.00) for CE, 0.67 (0.64–0.70) for LAA, and 0.70 (0.67–0.73) for SAO for unseen cases. ML-predicted IS subtypes yielded comparable subsequent stroke and all-cause mortality rates to the etiologically classified IS subtypes.

**Conclusion:**

This study highlighted substantial heterogeneity in prognosis of IS subtypes and utility of ML approaches for classification of IS cases with incomplete clinical investigations.

## Introduction

China has the highest prevalence of ischemic stroke (IS) cases worldwide with 24 million cases and 1 million deaths in 2019.^1,2^ Stroke is a heterogeneous disease, and reliable classification of IS cases by etiological subtypes (cardioaortic embolism (CE), large artery atherosclerosis (LAA), and small artery occlusion (SAO)/lacunar stroke) is required to assess prognosis and treatment recommendations. Validated algorithms for classification of IS cases by etiological subtypes include the Trial of Org 10172 in Acute Stroke Treatment (TOAST)^3^ and Causative Classification System for Ischemic Stroke (CCS),^4,5^ which classify IS cases using presenting symptoms, clinical signs, and imaging and vascular investigations. However, in population studies with incomplete investigations, the feasibility of using TOAST or CCS is limited, resulting in large proportions of unclassified IS cases.

The distributions of IS subtypes and their associated risk factors differ between Chinese and European populations,^6,7^ and also differ within China.^8^ IS cases in China are characterized by higher proportions of SAO, lower proportions of CE, and lower rates of atrial fibrillation (AF) compared with Western populations, in addition to substantial differences between regions within China.^6–8^ Previous studies of IS subtypes have been constrained by small numbers of cases, limited geographic diversity, and have been hospital-based rather than community-based.^6–8^

Supervised machine learning (ML) methods can be used to provide automated classification of IS subtypes using electronic health records^9–11^ and to predict stroke outcomes based on IS subtype.^12,13^ However, little is known about the utility of supervised ML for classifying IS subtypes in population studies with incomplete data. Since data on risk factors for stroke types are readily available in low-resource settings, ML approaches could enable probabilistic classification of IS cases in the absence of specialist imaging and vascular investigations.

The aims of this study were to determine the prevalence, characteristics, and prognosis of etiologically classified IS cases in a 9-year follow-up of the China Kadoorie Biobank (CKB), to use multiclass logistic regression (LR), a supervised ML method, to classify IS cases with incomplete investigations, and to compare the prevalence and prognosis of ML-predicted IS cases with etiologically classified IS cases.

## Methods

### Study design

Details of the design and methods used in the CKB have been previously described.^14,15^ Briefly, the CKB is a prospective study of 512,726 participants, aged 30–79 years, enrolled from five urban and five rural regions of China in 2004–2008, with follow-up data about stroke and death available until 1 January 2018. Information on health and lifestyle was collected by questionnaire. Physical measurements included height, weight, hip and waist circumference, bio-impedance, blood pressure, and heart rate (STable 1). Ethical approval for the CKB was obtained from the Oxford University Tropical Research Ethics Committee and the Chinese Center for Disease Control and Prevention Ethical Review Committee, and all participants provided written informed consent.

Ascertainment of non-fatal and fatal incident cases of stroke was undertaken by electronic linkage with death and disease registries and with health insurance (HI) agencies for all hospitalizations (over 97% of study participants had HI coverage at enrolment).^15^ All reported cases of stroke were classified according to International Classification of Diseases 10th Revision (ICD-10) by trained medical staff using bespoke standardization software that applied codes to original disease descriptions (including those pre-dating the adoption of ICD-10).^16^ All retrieved primary stroke cases underwent clinical adjudication, with review of medical records by local clinical specialists using a standardized electronic form (STable 2) including symptoms and signs, and brain imaging features for classification by TOAST^3^ and CCS^4,5^ (STable 3). Approximately, 20% of stroke cases reported to disease registries or HI agencies were fatal. However, it was rarely possible to obtain medical records for hospitalized stroke cases reported to death registries, particularly in rural areas where many participants died at home and consequently cases reported only from this source were not retrieved for adjudication. In addition, cases of subsequent hospitalized stroke were not adjudicated. However, a validation study of ~40,000 stroke cases retrieved by CKB revealed a reporting accuracy of 93% for IS.^17^

IS cases were classified using the following ICD-10 codes: I63 for IS (LACI (lacunar stroke), non-LACI); I61 for intracerebral hemorrhage; I60 for subarachnoid hemorrhage; I64 for unspecified stroke types; or R90.8 for silent cerebral infarcts that is imaging-detected cerebral infarctions without acute focal dysfunction (adopting the code for “Other abnormal findings on diagnostic imaging of central nervous system”). Following exclusion of participants with prior stroke or transient ischemic attack (TIA), 22,216 adjudication-confirmed cases of primary IS were classified using a modified CCS algorithm into etiological IS subtypes with confidence levels of “evident,” “probable,” “possible,” or “undetermined” cases. The adjudication form did not permit the classification of cases as “other determined etiology.” Additional details of the implementation of the modified CCS algorithm using CKB variables are provided in SMethods 1. “True” cases of each IS subtype were defined as those classified into evident and probable IS subtypes. Incompletely investigated cases were defined as strokes with missing brain imaging, cardiac tests (neither echocardiography (Echo) nor electrocardiogram (ECG) testing), or vascular imaging.

### Statistical analysis

The analyses were restricted to the first 9 years of follow-up of individuals with no prior stroke or TIA at enrolment (205,293 men, 298,549 women) in the CKB. Baseline characteristics were compared between individuals with incident IS subtypes of CE, LAA, or SAO; those with undetermined IS subtypes; and those without stroke of any type during follow-up. The cumulative rates of subsequent stroke (>28 days after initial stroke) of any type and all-cause mortality rate following a first IS event were compared by IS subtypes. Cumulative mortality after first incident stroke was calculated as one minus Kaplan–Meier survival probability with 95% confidence intervals (CIs) estimated using Greenwood’s Exponential formula. The cumulative subsequent stroke rates and corresponding 95% CIs were calculated by the cumulative incidence function, treating death from any cause as a competing risk.^18^

An 85:15 random split of evident and probable cases of CE, LAA, and SAO was used to generate a training set and held-out test set. Using the training set, we developed a multiclass LR model to classify IS cases based on baseline risk factors, in addition to screening for cardioaortic sources of embolism^4,5^ (STables 1–2 and SMethods 2). Feature selection from among 135 available input variables for the multiclass classifier (listed in STables 1–2) was performed using L1 regularization to maximize area under the receiver-operating characteristic curve (AUC). Regularization strength was optimized using fivefold cross-validation within the training set, with the tuned regularization strength used to fit the final LR model on all training data.^19^ The LR model was evaluated on IS cases with evident and probable etiology in the test set using AUC, positive predictive value (PPV), sensitivity, F1 score (the harmonic mean of PPV and sensitivity), and accuracy. The model was then applied to incompletely investigated IS cases with undetermined etiology and evaluated by comparing outcomes of ML-classified cases of each IS subtype with those of “true” cases with determined etiology (an established method of validating subtype endpoints in the absence of ground truth).^20^ Sensitivity analyses applied the model to IS cases with possible etiology and excluded cases with silent cerebral infarcts, which are included in the revised ICD-11, but not the ICD-10 definition of stroke.^16,21^ Additional details of the LR model development and evaluation are provided in SMethods 2. Analyses were performed using Python version 3.7.0. Kaplan–Meier estimates, including 95% CIs, were implemented using the lifelines package^22^ version 0.21.1, and the multiclass LR model was implemented using the scikit-learn toolkit^23^ version 0.19.2. Cumulative incidence of competing risks was estimated using the cmprsk package version 2.2-11 in R.^24^

### Results

**Figure 1.**
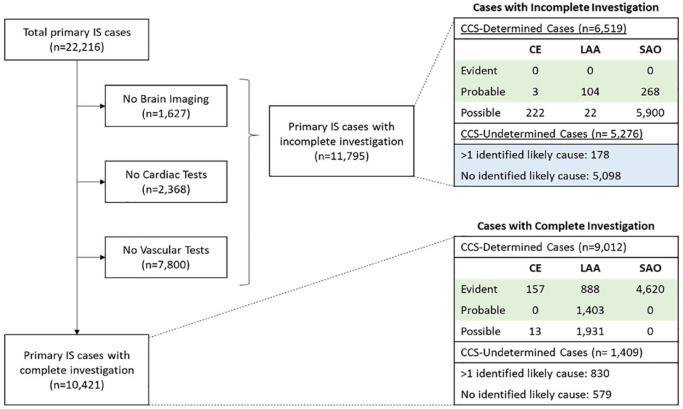
Flowchart of investigations for etiologically classified IS cases in the China Kadoorie Biobank. IS: ischemic stroke; CE: cardioaortic embolism; LAA: large artery atherosclerosis; SAO: small artery occlusion; CCS: Causative Classification System for Ischemic Stroke. Green cells indicate evident and probable cases included in the analyses of each IS subtype. Blue cells indicate incompletely investigated cases with undetermined etiology, classified in the present study using a novel machine learning approach.

Among 22,216 primary IS cases, 70% had a determined causative subtype, 48% (n = 7443) of which were classified as having evident or probable etiology (Figure 1). Overall, 91% of IS cases had brain imaging, 87% had at least one cardiac test (Echo or ECG), and 51% had at least one vascular test, resulting in 11,795 IS cases with incomplete investigations (Figure 1, STable 4). Approximately, 6% of ECG recordings used 24-h Holter monitoring. Importantly, 5276 (45%) incompletely investigated cases had undetermined etiology, representing 24% of all primary IS cases that may benefit from a probabilistic risk factor-based classification of IS subtypes with incomplete investigations. Among individuals with a primary IS case, the median follow-up time was 9.0 years from recruitment and 3.8 years from the time of first incident IS, with only 0.2% of individuals lost to follow-up.

**Figure 2.**
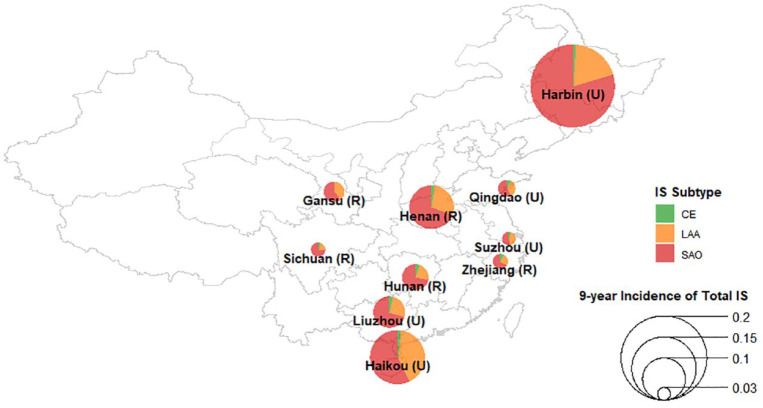
Proportions of etiologically classified IS cases by area in China. IS: ischemic stroke; CE: cardioaortic embolism; LAA: large artery atherosclerosis; SAO: small artery occlusion. Proportions of each IS subtype were calculated using cases with evident and probable determined etiology in each area (U: urban area; R: rural area). The size of each pie represents area-specific 9-year incidence of total IS, including cases with both determined and undetermined etiologies.

Among the evident and probable IS subtypes, 2% (n = 160) had CE, 32% (n = 2395) had LAA, and 66% (n = 4888) had SAO (Table 1). Overall, individuals with IS subtypes were older and more likely to be men, or have diabetes and hypertension, or a prior coronary heart disease (CHD), or have higher levels of body mass index (BMI) than those without stroke of any type (n = 445,246) (Table 1). Baseline risk factors were similar for different IS subtypes, but history of CHD and cardioaortic sources of embolism were more common in CE, while individuals with SAO were more likely to be women and to live in urban areas. The distribution of IS subtypes differed substantially by geographic region (Figure 2), with the 9-year incidence of IS varying by greater than sixfold and the ratio of SAO-to-LAA cases also varying substantially by geographic region.

**Table 1.** Baseline characteristics of participants with etiologically classified ischemic stroke subtypes.

	CE (n = 160)	LAA (n = 2395)	SAO (n = 4888)	Undetermined (incomplete investigation) (n = 5276)	No stroke^a^ (n = 445,246)
Demographic factors					
Age, mean (SD) (years)	61.9 (9.4)	59.9 (8.9)	58.7 (9.5)	59.0 (9.8)	50.9 (10.3)
Female (%)	46.9	46.3	59.1	54.9	59.7
Urban (%)	59.4	51.4	64.4	41.4	43.0
Medical history and health status (%)					
Diabetes mellitus	10.6	14.5	12.5	13.3	4.9
Coronary heart disease	14.4	7.8	7.4	7.2	2.2
Hypertension	59.4	61.9	54.6	59.0	30.6
Blood pressure drugs	29.4	24.0	21.5	23.1	9.2
Cardioaortic source of cerebral embolism^b^	100.0	4.1	0.3	3.4	—
Physical measurements, mean (SD)					
SBP (mmHg)	141.9 (25.6)	144.7 (23.8)	139.9 (23.1)	142.9 (24.5)	129.3 (20.2)
DBP (mmHg)	80.8 (13.5)	82.2 (12.6)	81.1 (12.2)	81.2 (12.3)	77.2 (10.8)
BMI (kg/m^2^)	24.8 (3.8)	24.2 (3.3)	24.6 (3.5)	24.4 (3.5)	23.6 (3.3)
Lifestyle factors (%)					
Male ever-regular smokers	71.8	74.9	71.6	75.8	74.5
Female ever-regular smokers	2.7	4.9	5.7	4.5	2.9
Male ever-regular drinkers	38.8	42.1	44.3	41.5	41.9
Female ever-regular drinkers	2.7	2.2	2.9	2.5	2.9

CE: cardioaortic embolism; LAA: large artery atherosclerosis; SAO: small artery occlusion; SBP: systolic blood pressure; DBP: diastolic blood pressure; BMI: body mass index; SD: standard deviation; CCS: Causative Classification System for Ischemic Stroke.

Evident and probable cases of CE, LAA, and SAO with complete or incomplete investigation are included. Diabetes mellitus and coronary heart disease physician-diagnosed and self-reported at baseline. Hypertension defined as SBP >140 mmHg, DBP >90 mmHg, or use of antihypertensive medication. Ever-regular smokers include current- and ex-regular smokers. Ever-regular drinkers include current-regular, ex-regular, and reduced-intake alcohol drinkers.

a Excludes hospital-reported stroke of any type (n = 58,596).

b Cardioaortic sources of cerebral embolism include those with high and low primary risk recognized by CCS and were identified from retrieved hospital stroke records rather than at baseline.

**Figure 3.**
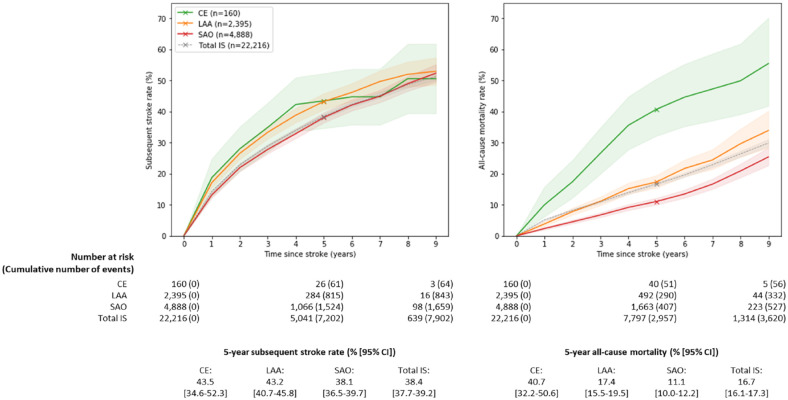
Estimated cumulative subsequent stroke cases and all-cause mortality rates following first incident etiologically classified IS cases. IS: ischemic stroke; CE: cardioaortic embolism; LAA: large artery atherosclerosis; SAO: small artery occlusion; CI: confidence interval. Evident and probable cases of CE, LAA, and SAO were included. Total IS: total ischemic stroke including cases with both determined and undetermined etiologies.

Importantly, CE accounted for only a small proportion of IS cases in China. In contrast, CE had the highest 5-year subsequent stroke and all-cause mortality rates (43.5% [95% CI: 34.6–52.3%] and 40.7% [95% CI: 32.2–50.6%], respectively), followed by LAA (43.2% [95% CI: 40.7–45.8%] and 17.4% [95% CI: 15.5–19.5%]) and SAO (38.1% [95% CI: 36.5–39.7%] and 11.1% [95% CI: 10.0–12.2%]) (Figure 3). All differences observed between the IS subtypes were statistically significant except for differences in the 5-year subsequent stroke rates for CE and LAA (due in part to the low number of CE cases) (Figure 3). Overall, the 5-year subsequent stroke rates and all-cause mortality rates for IS were 38.4% [37.7–39.2%] and 16.7% [16.1–17.3%], respectively. Subsequent stroke types following each of the IS subtypes were chiefly ischemic (91–94%) with only 5–6% being hemorrhagic (STable 5).

**Figure 4.**
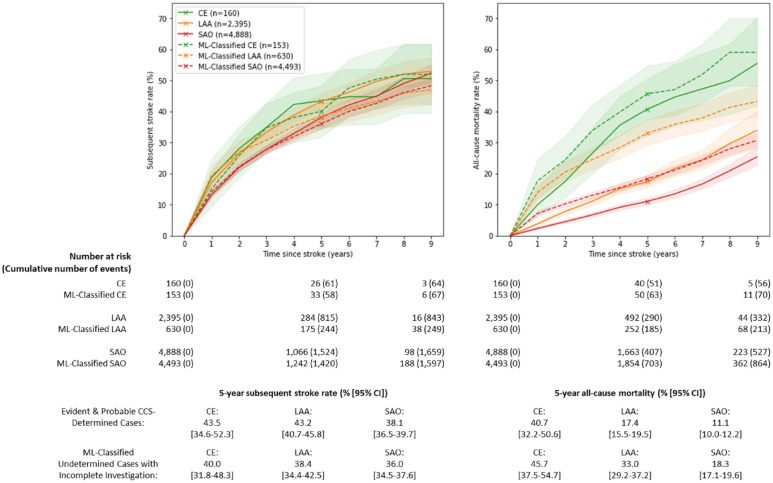
Subsequent stroke and all-cause mortality rates following first incident IS subtypes ascertained by (i) modified CCS (solid lines) and (ii) an ML approach for classifying IS cases with incomplete clinical investigation (dashed lines). IS: ischemic stroke; CE: cardioaortic embolism; LAA: large artery atherosclerosis; SAO: small artery occlusion; CCS: Clinical Causative Classification for Ischemic Stroke; ML: machine learning. Solid lines include etiologically classified evident and probable cases of CE, LAA, and SAO. Dashed lines include ML-classified IS cases with undetermined etiology and missing brain imaging, cardiac tests, or vascular tests.

The multiclass LR model yielded good overall discrimination of the unseen IS cases with evident and probable etiology in the test set (AUC: 0.80 [95% CI: 0.78–0.83]). There was near-perfect discrimination of CE (AUC: 0.99 [95% CI: 0.99–1.00]) due to the fact that findings of a cardioaortic source of embolism are nearly perfectly indicative of etiologically classified CE (SFigure 1). More modest discrimination was achieved for LAA (AUC: 0.67 [95% CI: 0.64–0.70]) and SAO (AUC: 0.70 [95% CI: 0.67–0.73]) (SFigure 1). The overall accuracy of predictions was 69% [95% CI: 0.67–0.71]. Model performance for the IS cases with possible etiology was comparable with slightly lower AUC scores (CE: 1.00 [1.00–1.00]; LAA: 0.59 [0.57–0.60]; SAO: 0.64 [0.63–0.66]) (SFigure 2). The chief variables required to identify CE included any cardioaortic sources of embolism (STable 6). Features that were important for distinguishing LAA and SAO included region, sex, access to HI, and family history of stroke (STable 6). After applying these features to IS cases with undetermined etiology, the ML-classified IS (3% CE, 12% LAA, and 85% SAO) had a comparable prognosis to the “true” evident and probable cases of each subtype. ML-classified CE cases had the highest 5-year subsequent stroke and all-cause mortality rates (40.0% [31.8–48.3%] and 45.7% [37.5–54.7%], respectively), followed by LAA (38.4% [34.4–42.5%] and 33.0% [29.2–37.2%]) and SAO (36.0% [34.5–37.6%] and 18.3% [17.1–19.6%]) (Figure 4). However, mortality rates subsequent to an ML-classified incompletely investigated IS were consistently higher than the “true” cases of each IS subtype.

Silent cerebral infarcts had lower 5-year subsequent stroke rates (33.7% [31.9–35.5%]) and all-cause mortality (10.1% [9.0–11.4%]) than symptomatic strokes of all IS subtypes (SFigure 5). Sensitivity analyses conducted after excluding cases with silent cerebral infarcts yielded broadly similar results to the overall analyses of all IS cases (STables 7–8, SFigures 3–6).

## Discussion

Overall, about 7% of participants had a primary IS case during the first 9 years of follow-up. The prognosis of IS cases was poor, with 38% having a subsequent stroke (>90% IS) within 5 years and 17% dying within 5 years, consistent with findings from previous analyses of cumulative mortality and subsequent vascular events following incident stroke reported by CKB.^25^ Prognosis also differed substantially by IS subtypes, with CE having the worst outcomes, followed by LAA and SAO, consistent with results of previous smaller studies.^7,26,27^

The study demonstrated the utility of multiclass LR to provide IS subtype classifications for IS cases with undetermined etiology. While the AUCs were too modest for making reliable clinical predictions for individuals, the use of ML did reveal important insights into IS subtypes in population studies. The ML-classified IS cases yielded concordant subsequent stroke rates and generally concordant, albeit higher, all-cause mortality rates compared with etiologically classified IS cases. The higher mortality rates for ML-classified IS cases may reflect regional differences in use of clinical investigations, including vascular imaging, and use of preventive treatments across China. For example, incompletely investigated cases were more common in rural areas than in urban areas (59% vs 48% of IS cases, respectively), tracking with higher mortality rates 5 years after first IS (20.0% vs 14.2%, respectively).

The proportions of IS cases in China differed substantially from those in Western populations.^28^ The proportion of CE cases in China was 18-fold lower (2% vs 36%), while the proportion of LAA was comparable (32% vs 33%), and the proportion of SAO was twofold greater in China than in Western populations (66% vs 31%). The findings of low rates of CE in CKB are consistent with previous estimates of the prevalence of CHD (8–15%) and AF (4–10%) among IS cases in China,^6–8,29,30^ which are lower than in the United States^20^ and Europe^27^ (CHD: 20–39%, AF: 26–27%). However, the low rates of CE (2%) reported in the present study compared with previous studies in China (8–14%) may reflect underreporting of AF in rural areas of China with more limited resources, which were not included in previous studies.^7,8^ It is likely that the proportion of CE subtypes reinforced by the diagnosis of AF was underestimated by the low utilization of prolonged cardiac (24-h Holter) monitoring in hospitalized IS cases.

The present had several strengths, including the prospective study design, large number of participants, detailed data on stroke subtypes, and prolonged follow-up. Nevertheless, the study also had several limitations. First, while the study was designed to examine the causes of disease in diverse regions of China, the CKB study is not representative of the overall Chinese population. Second, baseline data were not available on AF or blood lipids on all participants. Third, the inclusion of region in our classification model may limit the generalizability of this approach to other regions in China. Fourth, while one-fifth of stroke cases reported to disease registries and HI agencies were fatal, those reported exclusively to death registries were not retrieved for adjudication as medical notes were rarely obtainable. Since these unadjudicated stroke cases were not further classified, all-cause mortality rates for all IS subtypes may have been underestimated. Finally, subsequent hospitalized cases of stroke were not adjudicated, resulting in potential over- or underestimation of subsequent stroke and all-cause mortality rates and misclassification of IS subtypes. However, CKB retrieved medical records on 38,823 stroke cases and demonstrated a diagnostic accuracy of 81% for all strokes and of 79% for combined IS subtypes, which increased to 94% and 93%, respectively, when silent cerebral infarcts detected on brain imaging—defined as IS in ICD-11—were also included.^17^

Overall, the present study highlights differences in the distribution and prognosis of IS subtypes in Chinese adults and the utility of using supervised ML methods to classify IS cases of undetermined etiology into predicted IS subtypes for population-level analyses. Such classifications could improve the assessment of IS etiology and prognosis in population studies and personalized use of medication tailored to IS subtypes in clinical practice.

## Supplemental Material

sj-docx-1-wso-10.1177_17474930231162265 – Supplemental material for Heterogeneity in the diagnosis and prognosis of ischemic stroke subtypes: 9-year follow-up of 22,000 cases in Chinese adults Click here for additional data file. Supplemental material, sj-docx-1-wso-10.1177_17474930231162265 for Heterogeneity in the diagnosis and prognosis of ischemic stroke subtypes: 9-year follow-up of 22,000 cases in Chinese adults by Matthew Chun, Haiqiang Qin, Iain Turnbull, Sam Sansome, Simon Gilbert, Alex Hacker, Neil Wright, Tingting Zhu, David Clifton, Derrick Bennett, Yu Guo, Pei Pei, Jun Lv, Canqing Yu, Ling Yang, Liming Li, Yan Lu, Zhengming Chen, Benjamin J Cairns, Yiping Chen and Robert Clarke in International Journal of Stroke
