# Supplementary material for: Heterogeneity in the diagnosis and prognosis of ischemic stroke subtypes: 9-year follow-up of 22,000 cases in Chinese adults
Source: Int J Stroke. 2023 Mar 16;18(7):847–55. doi: 10.1177/17474930231162265 (PMC10374992; doi:10.1177/17474930231162265)
Supplement: sj-docx-1-wso-10.1177_17474930231162265 – Supplemental material for Heterogeneity in the diagnosis and prognosis of ischemic stroke subtypes: 9-year follow-up of 22,000 cases in Chinese adults [file sj-docx-1-wso-10.1177_17474930231162265.docx]

**Supplementary Materials for**

“Heterogeneity in diagnosis and prognosis of ischaemic stroke subtypes:

9-year follow-up of 22000 cases in Chinese adults”

[**Members of the China Kadoorie Biobank Collaborative Group** 3](#_Toc119920711)

[**SMethods 1.** Implementation of Causative Classification System for Ischemic Stroke (CCS) in the China Kadoorie Biobank (CKB) 4](#_Toc119920712)

[**SMethods 2.** Details of multiclass logistic regression (LR) model development and evaluation 6](#_Toc119920713)

[**SFigure 1.** Performance of multiclass logistic regression (LR) model on evident and probable cases of each ischaemic stroke (IS) subtype in test set. Macro-averaged metrics represent the average of the metrics across IS subtypes, giving equal weight to CE, LAA, and SAO. Weighted average metrics represent the average of the metrics across IS subtypes weighting the metrics for CE, LAA, and SAO by the respective prevalence of each IS subtype. 9](#_Toc119920714)

[**SFigure 2.** Performance of multiclass logistic regression (LR) model on possible cases of each ischaemic stroke (IS) subtype. Macro-averaged metrics represent the average of the metrics across IS subtypes, giving equal weight to CE, LAA, and SAO. Weighted average metrics represent the average of the metrics across IS subtypes weighting the metrics for CE, LAA, and SAO by the respective prevalence of each IS subtype. 10](#_Toc119920715)

[**SFigure 3.** Performance of multiclass logistic regression (LR) model on evident and probable cases of each ischaemic stroke (IS) subtype in test set (excluding silent cerebral infarcts). Macro-averaged metrics represent the average of the metrics across IS subtypes, giving equal weight to CE, LAA, and SAO. Weighted average metrics represent the average of the metrics across IS subtypes weighting the metrics for CE, LAA, and SAO by the respective prevalence of each IS subtype. 11](#_Toc119920716)

[**SFigure 4.** Performance of multiclass logistic regression (LR) model on possible cases of each ischaemic stroke (IS) subtype (excluding silent cerebral infarcts). Macro-averaged metrics represent the average of the metrics across IS subtypes, giving equal weight to CE, LAA, and SAO. Weighted average metrics represent the average of the metrics across IS subtypes weighting the metrics for CE, LAA, and SAO by the respective prevalence of each IS subtype. 12](#_Toc119920717)

[**SFigure 5.** Estimated cumulative subsequent stroke and all-cause mortality rates after first ischaemic stroke (IS) of (i) different subtypes (excluding silent cerebral infarcts) and (ii) silent cerebral infarcts. 13](#_Toc119920718)

[**SFigure 6.** Subsequent stroke and all-cause mortality rates of different ischaemic stroke subtypes for (i) cases with evident and probable determined aetiology and (ii) machine learning (ML)-classified strokes with undetermined aetiology due to incomplete investigation (excluding silent cerebral infarcts). 14](#_SFigure_6._Subsequent)

[**STable 1.** List of all baseline CKB variables considered in the present study. 15](#_Toc119920720)

[**STable 2:** Copy of adjudication form used by Chinese Clinicians to classify stroke types 21](#_Toc119920721)

[**STable 3:** Key variables collected from medical records for classification of IS subtypes using CCS. 27](#_Toc119920722)

[**STable 4:** Breakdown of clinical investigations for aetiologically-classified ischaemic stroke (IS) subtypes of evident or probable confidence level and ML-classified IS subtypes. 28](#_Toc119920723)

[**STable 5:** Breakdown of second strokes by pathological types following a first stroke of different ischaemic stroke (IS) subtypes. 29](#_Toc119920724)

[**STable 6:** Top risk factors for classifying ischaemic stroke (IS) subtypes. 30](#_Toc119920725)

[**STable 7:** Breakdown of primary ischaemic stroke (IS) cases with separation of silent and non-silent strokes. 31](#_Toc119920726)

[**STable 8:** Top risk factors for classifying ischaemic stroke (IS) subtypes (excluding silent cerebral infarcts). 32](#_Toc119920727)

**Supplementary** [**References** 33](#_Toc119920728)

# Members of the China Kadoorie Biobank Collaborative Group

**International Steering Committee**: Junshi Chen, Zhengming Chen (PI), Robert Clarke, Rory Collins, Yu Guo, Liming Li (PI), Chen Wang, Jun Lv, Richard Peto, Robin Walters.

**International Co-ordinating Centre, Oxford**: Daniel Avery, Derrick Bennett, Ruth Boxall, Sushila Burgess, Ka Hung Chan, Yiping Chen, Zhengming Chen, Johnathan Clarke; Robert Clarke, Huaidong Du, Ahmed Edris Mohamed, Hannah Fry, Simon Gilbert, Mike Hill, Pek Kei Im, Andri Iona, Maria Kakkoura, Christiana Kartsonaki, Hubert Lam, Kuang Lin, Mohsen Mazidi, Iona Millwood, Sam Morris, Qunhua Nie, Alfred Pozarickij, Paul Ryder, Saredo Said, Dan Schmidt, Paul Sherliker, Becky Stevens, Iain Turnbull, Robin Walters, Lin Wang, Neil Wright, Ling Yang, Xiaoming Yang, Pang Yao.

**National Co-ordinating Centre, Beijing**: Yu Guo, Xiao Han, Can Hou, Qingmei Xia, Chao Liu, Jun Lv, Pei Pei, Canqing Yu.

**10 Regional Co-ordinating Centres:
Guangxi** Provincial CDC: Naying Chen, Duo Liu, Zhenzhu Tang. Liuzhou CDC: Ningyu Chen, Qilian Jiang, Jian Lan, Mingqiang Li, Yun Liu, Fanwen Meng, Jinhuai Meng, Rong Pan, Yulu Qin, Ping Wang, Sisi Wang, Liuping Wei, Liyuan Zhou. **Gansu** Provincial CDC: Caixia Dong, Pengfei Ge, Xiaolan Ren. Maiji CDC: Zhongxiao Li, Enke Mao, Tao Wang, Hui Zhang, Xi Zhang. **Hainan** Provincial CDC: Jinyan Chen, Ximin Hu, Xiaohuan Wang. Meilan CDC: Zhendong Guo, Huimei Li, Yilei Li, Min Weng, Shukuan Wu. **Heilongjiang** Provincial CDC: Shichun Yan, Mingyuan Zou, Xue Zhou. Nangang CDC: Ziyan Guo, Quan Kang, Yanjie Li, Bo Yu, Qinai Xu. **Henan** Provincial CDC: Liang Chang, Lei Fan, Shixian Feng, Ding Zhang, Gang Zhou. Huixian CDC: Yulian Gao, Tianyou He, Pan He, Chen Hu, Huarong Sun, Xukui Zhang. **Hunan** Provincial CDC: Biyun Chen, Zhongxi Fu, Yuelong Huang, Huilin Liu, Qiaohua Xu, Li Yin. Liuyang CDC: Huajun Long, Xin Xu, Hao Zhang, Libo Zhang. **Jiangsu** Provincial CDC: Jian Su, Ran Tao, Ming Wu, Jie Yang, Jinyi Zhou, Yonglin Zhou. Suzhou CDC: Yihe Hu, Yujie Hua, Jianrong Jin, Fang Liu, Jingchao Liu, Yan Lu, Liangcai Ma, Aiyu Tang, Jun Zhang. **Qingdao** Provincial CDC: Liang Cheng, Ranran Du, Ruqin Gao, Feifei Li, Shanpeng Li, Yongmei Liu, Feng Ning, Zengchang Pang, Xiaohui Sun, Xiaocao Tian, Shaojie Wang, Yaoming Zhai, Hua Zhang, Licang CDC: Wei Hou, Silu Lv, Junzheng Wang. **Sichuan** Provincial CDC: Xiaofang Chen, Xianping Wu, Ningmei Zhang, Weiwei Zhou. Pengzhou CDC: Xiaofang Chen, Jianguo Li, Jiaqiu Liu, Guojin Luo, Qiang Sun, Xunfu Zhong. **Zhejiang** Provincial CDC: Weiwei Gong, Ruying Hu, Hao Wang,Meng Wang, Min Yu. Tongxiang CDC: Lingli Chen, Qijun Gu, Dongxia Pan，Chunmei Wang, Kaixu Xie, Xiaoyi Zhang.

# **SMethods 1.** Implementation of Causative Classification System for Ischemic Stroke (CCS) in the China Kadoorie Biobank (CKB)

Primary cases of ischaemic stroke (IS) in CKB were classified using a web-based implementation of the Causative Classification System for Ischemic Stroke (CCS),^1,2^ which can be accessed at <https://ccs.mgh.harvard.edu/ccs_form.php>. The modified IS aetiological subtypes used were: large artery atherosclerosis (LAA); small artery occlusion (SAO); cardioaortic embolism (CE); and undetermined aetiology.

In CKB, adjudicators were asked to complete a standardised electronic form, which requested specific details on presenting symptoms and signs and diagnostic criteria, including imaging evidence of haemorrhage or ischaemia, laterality and anatomical location of any cerebral lesions, and additional evidence of extracranial and intracranial arterial disease or sources of embolism. Where necessary, collected variables were slightly adapted to be fitted to the CCS algorithm, as described in further detail below. In addition, CCS has certain limitations for use in East Asian cohorts, which are also discussed below.

Fitting of CKB Variables to CCS:

- **Brain Imaging:**  In CCS, a lacunar lesion is defined as being <20 mm in diameter, while in CKB, lacunar lesions were defined as being <15 mm in diameter. CKB also did not include imaging variables for internal watershed infarcts or temporally separate infarcts. While CKB had capacity to identify cases with multiple acute ischaemic lesions in both the left and right anterior or both the anterior and posterior circulations, these were not included for IS subtyping. Finally, in CCS, one criterion for a possible SAO is presentation of “a classical lacunar syndrome in the absence of imaging that is sensitive enough to detect small infarctions.” In CKB, all IS cases with computed tomography (CT) and/or magnetic resonance imaging (MRI) were included among cases with brain imaging, with no distinction made between the sensitivities of CT and MRI. Findings of small infarctions from both CT and MRI were included.
- **Vascular Imaging:** From collected CKB variables, the relationship of arterial disease to the anatomical site of infarct could not be determined with certainty, which is relevant in CCS for considering parent artery disease. Second, for simplicity, stenosis of <50% or ≥50% was considered if it was determined to be present in any artery (intracranial or extracranial). Third, details of plaque echogenicity in the extracranial arteries only (i.e., hypoechoic or mixed echoic findings) were used as surrogates of thrombus formation and ulceration.
- **Cardiac Investigations and Other Clinical Findings:** First, the CKB variable for systemic embolism, which differentiates “Probable” from “Possible” CE cases was selected in only 26 IS cases and may be underreported. Second, some high- and low- or uncertain risk cardioaortic sources of cerebral embolism considered by CCS were not included in the CKB standardised electronic adjudication form. Third, while prior history of IS, transient monocular blindness, or transient ischaemic attack can be influential in CCS, these were not appropriate in the present CKB analyses, which considered incident primary IS cases only, and excluded participants with prior history of stroke or transient ischaemic attack at baseline. Finally, the CKB standardised electronic adjudication form did not include any means to record other causes of multifocal ischaemic brain injury (e.g., vasculitides, vasculopathies, and haemostatic or haemodynamic disturbances), hence the “Other determined aetiology” subtype was not assigned to IS cases in the present study.

Limitations of Using CCS in CKB:

- **Stroke Risk Estimates:** In CCS, a 2% annual or 1-time primary stroke risk threshold was chosen to separate “evident” from “possible” mechanisms. This threshold was chosen because it is the approximate, annual, primary, ipsilateral stroke risk associated with asymptomatic carotid stenosis greater than 50%.^1^ However, these estimates are based on Western populations rather than East Asian cohorts and may vary for CKB. In addition, CCS uses an arbitrary cutoff value of 2 for the positive likelihood ratio at which the presence of a feature is considered for distinguishing the most likely aetiological mechanism. This arbitrary cutoff may also require adjustment for use in East Asian cohorts for similar reasons.

Summary

Overall in this 9-year follow-up of a prospective study of 512,726 Chinese adults, 22,216 incident IS cases, confirmed by clinical adjudication of medical records, were assigned subtypes using a modified Causative Classification System for Ischemic Stroke (CCS) (LAA: large artery atherosclerosis; SAO: small artery occlusion, CE: cardioaortic embolism; or undetermined aetiology) and classified by CCS as “evident”, “probable”, or “possible” IS cases. Cases with determined causative subtypes were defined as all those with “evident”, “probable”, and “possible” confidence levels, and included all determined cases with complete investigation (n = 9,012) and with incomplete investigation (n = 6,519). The total number of cases with determined causative subtypes was 70% of all primary IS cases (15,531 / 22,216).

# **SMethods 2.** Details of multiclass logistic regression (LR) model development and evaluation

Data Release

Data used in this study were from CKB version 17.02.

Data Preprocessing

*Training Set and Test Set Split*

Individuals with a recorded primary IS case of evident and probable determined aetiology (n=7,443) were divided into a training set and test set using a random 85%/15% training/test split and checked to ensure similar proportions of CE/LAA/SAO.

|  | **Training Set (n=6,326)**  **Number of individuals (%)** | **Test Set (n=1,117)**  **Number of individuals (%)** |
| --- | --- | --- |
| CE | 136 ( 2.1%) | 24 ( 2.1%) |
| LAA | 2,036 (32.2%) | 359 (32.1%) |
| SAO | 4,154 (65.7%) | 734 (65.7%) |

*Missing Values*

For the 7,443 individuals included in the modeling analyses, missing values were very rare among the CKB baseline variables and were imputed in both the training set and test set using the sex-specific means of the non-missing values in the training set. Of the 134 baseline variables considered in CKB (listed in STable 1), only 14 risk factors had missing values. 9 individuals (0.1%) had missing values for number of siblings and siblings’ medical history (stroke, heart attack, diabetes, and cancer); 9 individuals (0.1%) had missing values for mother’s medical history (stroke, heart attack, diabetes, and cancer); 18 individuals (0.2%) had missing values for father’s medical history (stroke, heart attack, diabetes, and cancer); and 4 individuals (<0.1%) had missing values for body fat percentage. In addition to mean imputation, 3 binary risk factor indicator variables were added as model inputs to represent whether or not an individual was missing medical history for their mother, father, or siblings, respectively.

As described in the main text, findings of cardioaortic sources of embolism^1,2^ from cardiac tests identified from hospital records (“ce_risk” variable in STable 2) were also included as inputs to the multiclass LR model. Although not recorded at baseline in CKB, potential sources of embolism were included as model inputs because they are important for distinguishing CE, the determinants were present prior to the onset of stroke, and they can be cost-effectively detected with Echo or ECG tests. This variable was coded as 0: no identified cardioembolic source (including due to lack of cardiac tests), 1: low- or uncertain risk cardioembolic source, or 2: high-risk cardioembolic source, in accordance with the cardioaortic sources of cerebral embolism recognised by CCS. This input variable did not include any missing values.^1,2^

Multiclass Logistic Regression (LR) Model Construction:

A multiclass LR model was derived using the LogisticRegressionCV function of the scikit-learn toolkit^3^ version 0.19.2. The LR classifier was trained to predict, using the CKB baseline variables and findings of cardioaortic sources of embolism, a probability that each individual’s first stroke would be CE, LAA, or SAO conditional on a first IS event occurring.

*Hyperparameter Tuning*

Hyperparameters were tuned to maximise area under the receiver-operating characteristic curve (AUC), a measure of discrimination performance ranging from 0 to 1, for each individual subtype using 5-fold cross validation within the training set in a “one-versus-rest” multiclass strategy. The L1 penalty was used for regularisation and feature selection, and the LR model was computed using the “liblinear” solver.

For the main model described in the manuscript, the final tuned values for the hyperparameter, “C” (describing the inverse of regularisation strength) were: 2.78 for CE, 0.36 for LAA, and 0.36 for SAO. Smaller values of “C” correspond to greater regularisation strength, which tends to shrink parameter estimates of the model to 0, resulting in a simpler model with fewer input variables. Tuning “C” can prevent overfitting of machine learning models.

For the sensitivity analysis model, which excluded silent cerebral infarction, a similar hyperparameter tuning process was implemented, and the final tuned values for “C” were: 0.36 for CE, 0.36 for LAA, and 0.36 for SAO.

Upon determining the final tuned values for “C” using 5-fold cross validation, the final trained LR model (including selected features) was determined by refitting the model on all training data using the tuned “C” values.

*Model Evaluation*

The trained LR model was first evaluated on IS cases with evident and probable aetiology in the unseen test set using AUC, positive predictive value (PPV), sensitivity, F1 score, and accuracy. The model was also similarly evaluated on IS cases with possible aetiology. A summary of each evaluation metric is provided below:

- AUC: A measure of discrimination performance, ranging from 0 to 1. An AUC of 0.5 represents the performance of a model that performs no better than randomly classifying IS subtypes. An AUC of 1.0 represents perfect discrimination.
  - AUC scores were calculated for each IS subtype using both “one-versus-one” and “one-versus-rest” strategies. In the “one-versus-one” strategy, the discrimination performance is measured for each possible pair of IS subtypes (CE and LAA, CE and SAO, LAA and SAO). In the “one-versus-rest” strategy, the discrimination performance is measured once for each IS subtype (e.g., CE and non-CE, LAA and non-LAA, SAO and non-SAO.
- PPV: A metric representing how likely it is for an IS case to truly be a particular IS subtype given that the LR model classifies it as that subtype. Values range from 0 to 1, with 1 representing perfect PPV.
- Sensitivity: A metric representing the proportion of cases of a particular IS subtype that are correctly classified by the LR model as that subtype. Values range from 0 to 1, with 1 representing perfect sensitivity.
- F1 score: A metric representing the harmonic mean of the LR model’s PPV and sensitivity, capturing the tradeoff between these metrics. Values range from 0 to 1, with 1 representing a perfect F1 score.
- Accuracy: A metric representing the proportion of correctly classified IS subtypes. Values range from 0 to 1, with 1 representing perfect accuracy.

Finally, the model was applied to incompletely investigated IS cases with undetermined aetiology, and evaluated by comparing subsequent stroke and all-cause mortality rates of ML-classified cases of each IS subtype with those of “true” cases with determined aetiology (an established method of validating subtype endpoints in the absence of ground truth).^4^

**
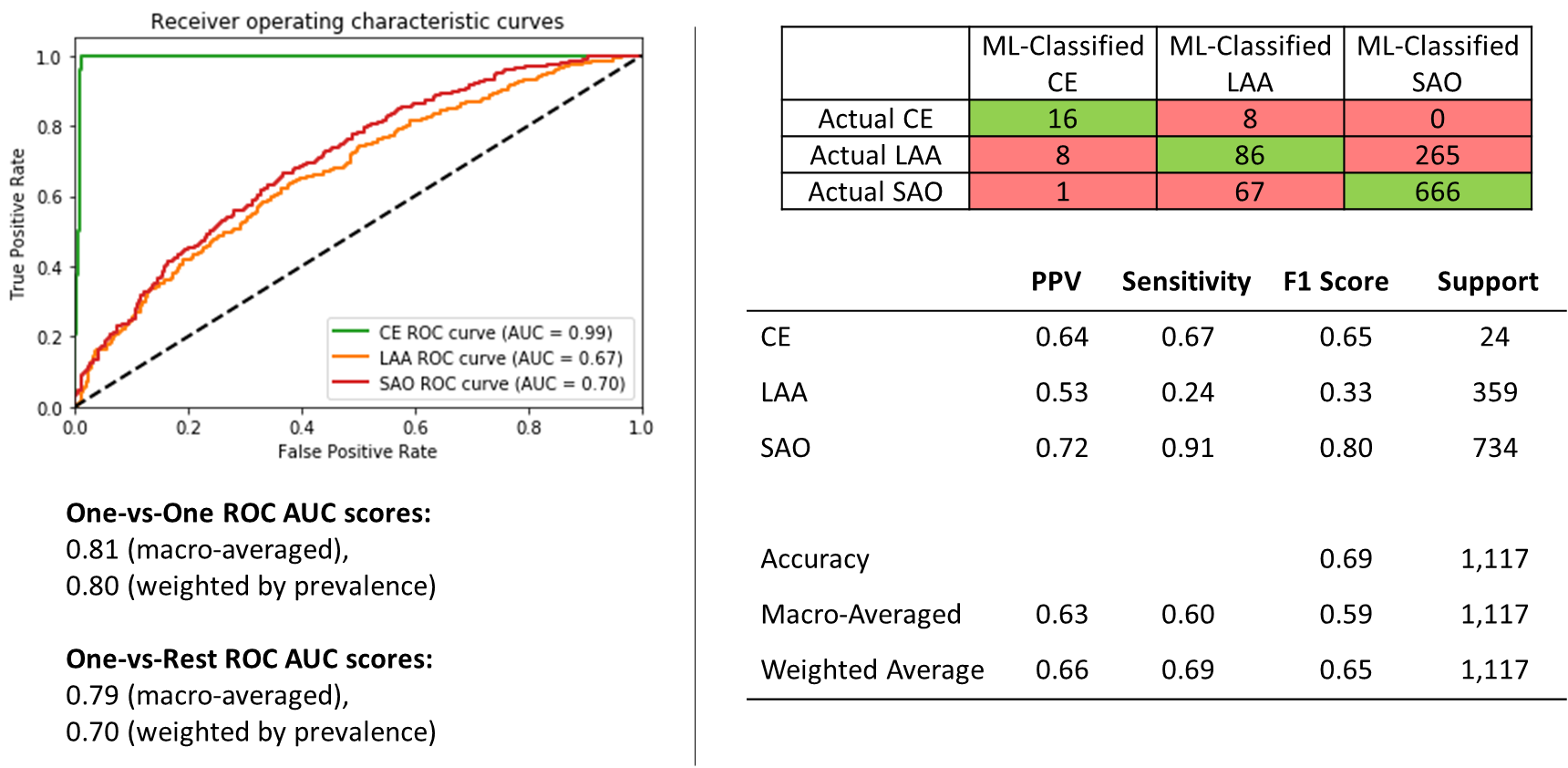
**

# **SFigure 1.** Performance of multiclass logistic regression (LR) model on evident and probable cases of each ischaemic stroke (IS) subtype in test set. Macro-averaged metrics represent the average of the metrics across IS subtypes, giving equal weight to CE, LAA, and SAO. Weighted average metrics represent the average of the metrics across IS subtypes weighting the metrics for CE, LAA, and SAO by the respective prevalence of each IS subtype.

CE: Cardioaortic embolism. LAA: Large artery atherosclerosis. SAO: Small artery occlusion. ML: machine learning. ROC AUC: Area under the receiver operating characteristic curve.

**
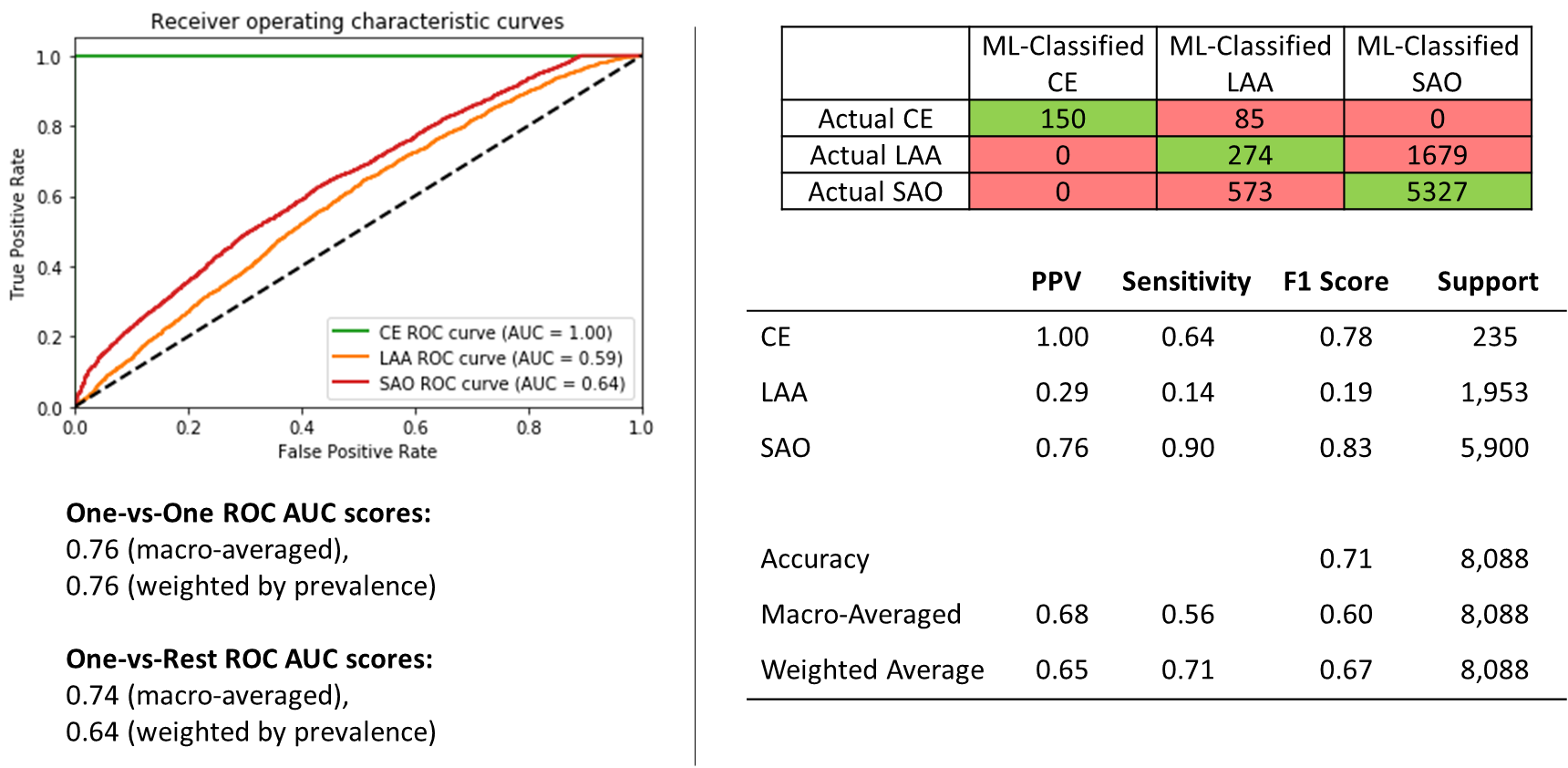
**

# **SFigure 2.** Performance of multiclass logistic regression (LR) model on possible cases of each ischaemic stroke (IS) subtype. Macro-averaged metrics represent the average of the metrics across IS subtypes, giving equal weight to CE, LAA, and SAO. Weighted average metrics represent the average of the metrics across IS subtypes weighting the metrics for CE, LAA, and SAO by the respective prevalence of each IS subtype.

CE: Cardioaortic embolism. LAA: Large artery atherosclerosis. SAO: Small artery occlusion. ML: machine learning. ROC AUC: Area under the receiver operating characteristic curve.

**
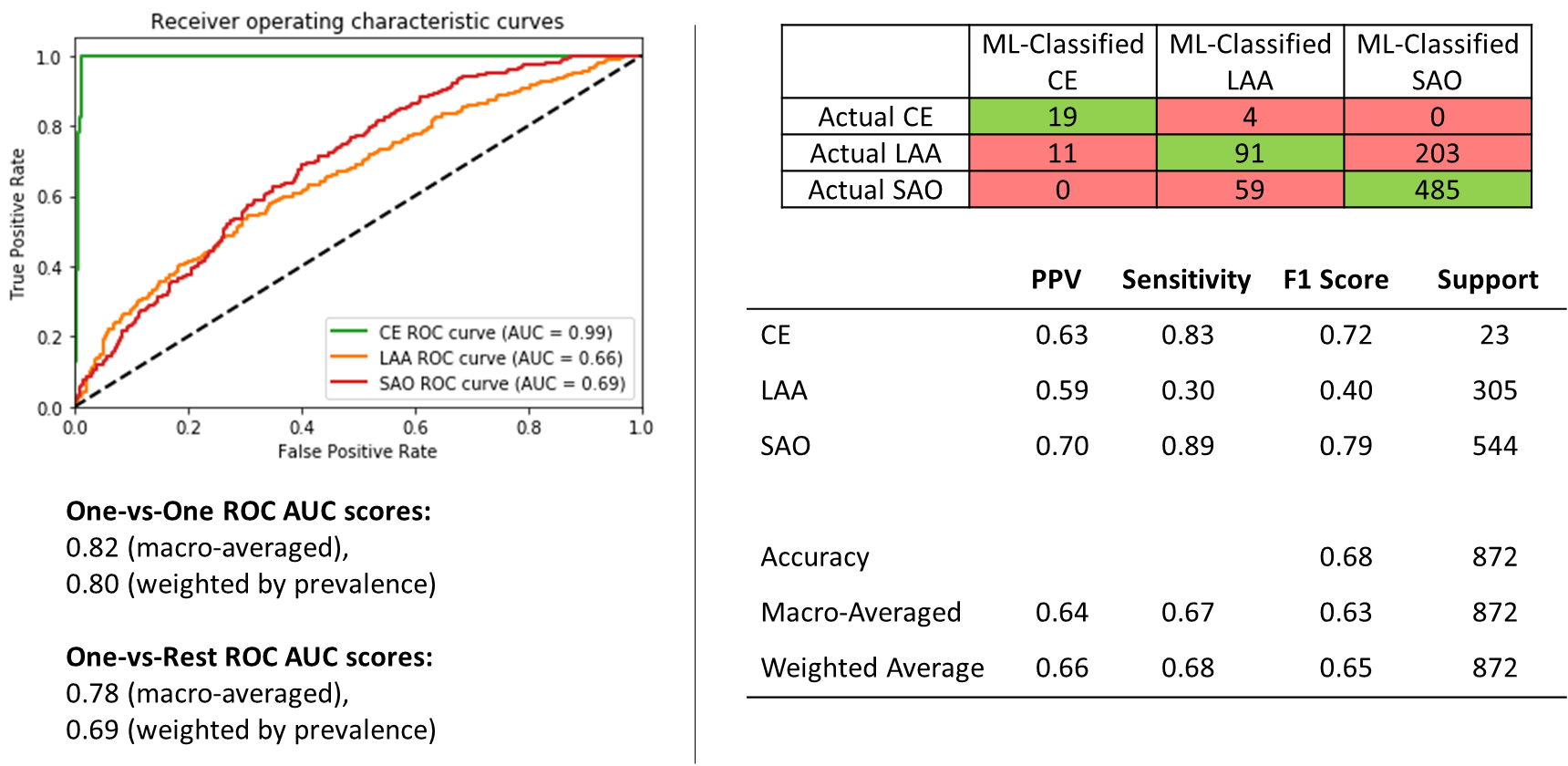
**

# **SFigure 3.** Performance of multiclass logistic regression (LR) model on evident and probable cases of each ischaemic stroke (IS) subtype in test set (excluding silent cerebral infarcts). Macro-averaged metrics represent the average of the metrics across IS subtypes, giving equal weight to CE, LAA, and SAO. Weighted average metrics represent the average of the metrics across IS subtypes weighting the metrics for CE, LAA, and SAO by the respective prevalence of each IS subtype.

CE: Cardioaortic embolism. LAA: Large artery atherosclerosis. SAO: Small artery occlusion. ML: machine learning. ROC AUC: Area under the receiver operating characteristic curve.

**
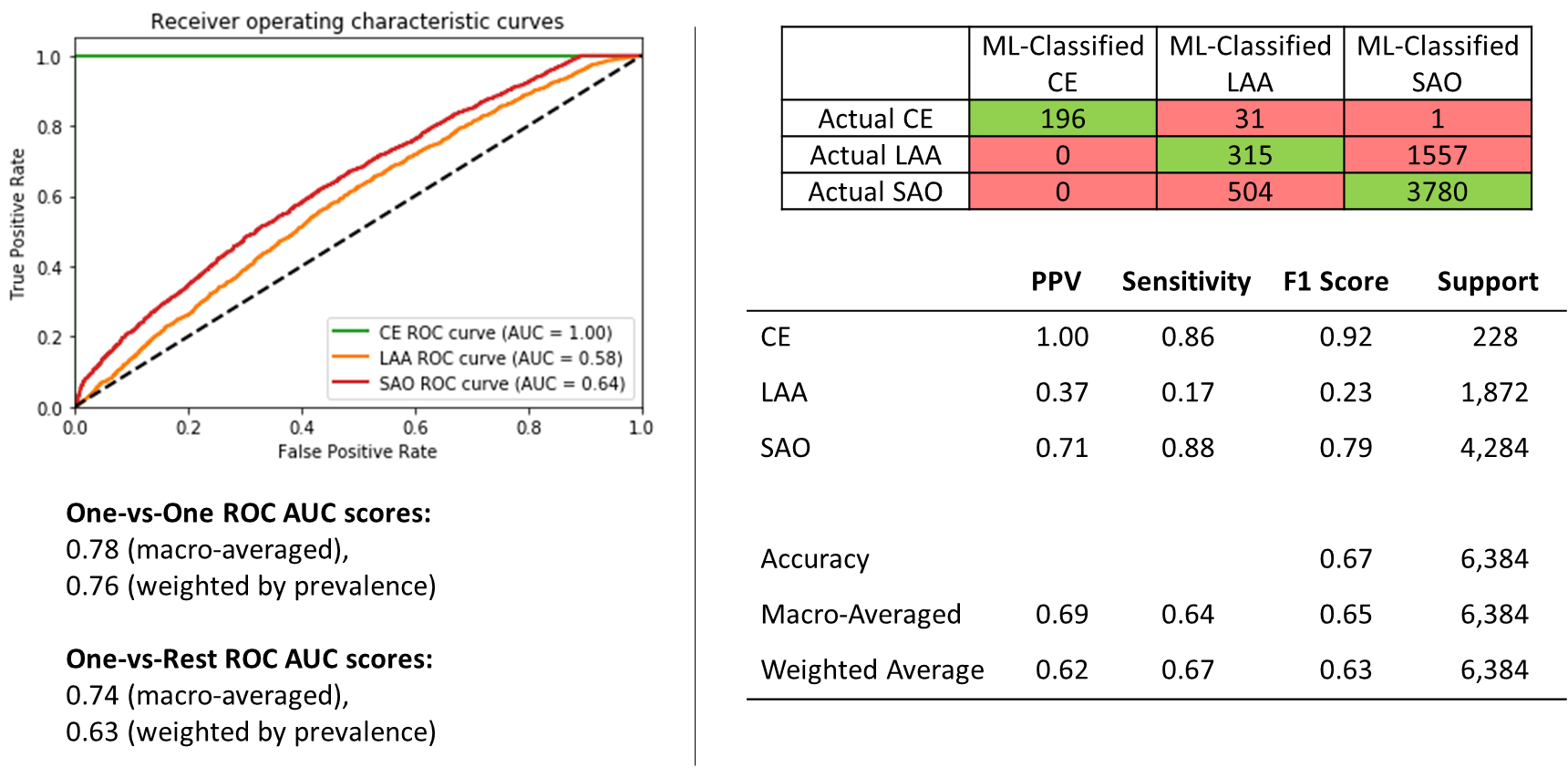
**

# **SFigure 4.** Performance of multiclass logistic regression (LR) model on possible cases of each ischaemic stroke (IS) subtype (excluding silent cerebral infarcts). Macro-averaged metrics represent the average of the metrics across IS subtypes, giving equal weight to CE, LAA, and SAO. Weighted average metrics represent the average of the metrics across IS subtypes weighting the metrics for CE, LAA, and SAO by the respective prevalence of each IS subtype.

CE: Cardioaortic embolism. LAA: Large artery atherosclerosis. SAO: Small artery occlusion. ML: machine learning. ROC AUC: Area under the receiver operating characteristic curve.

**
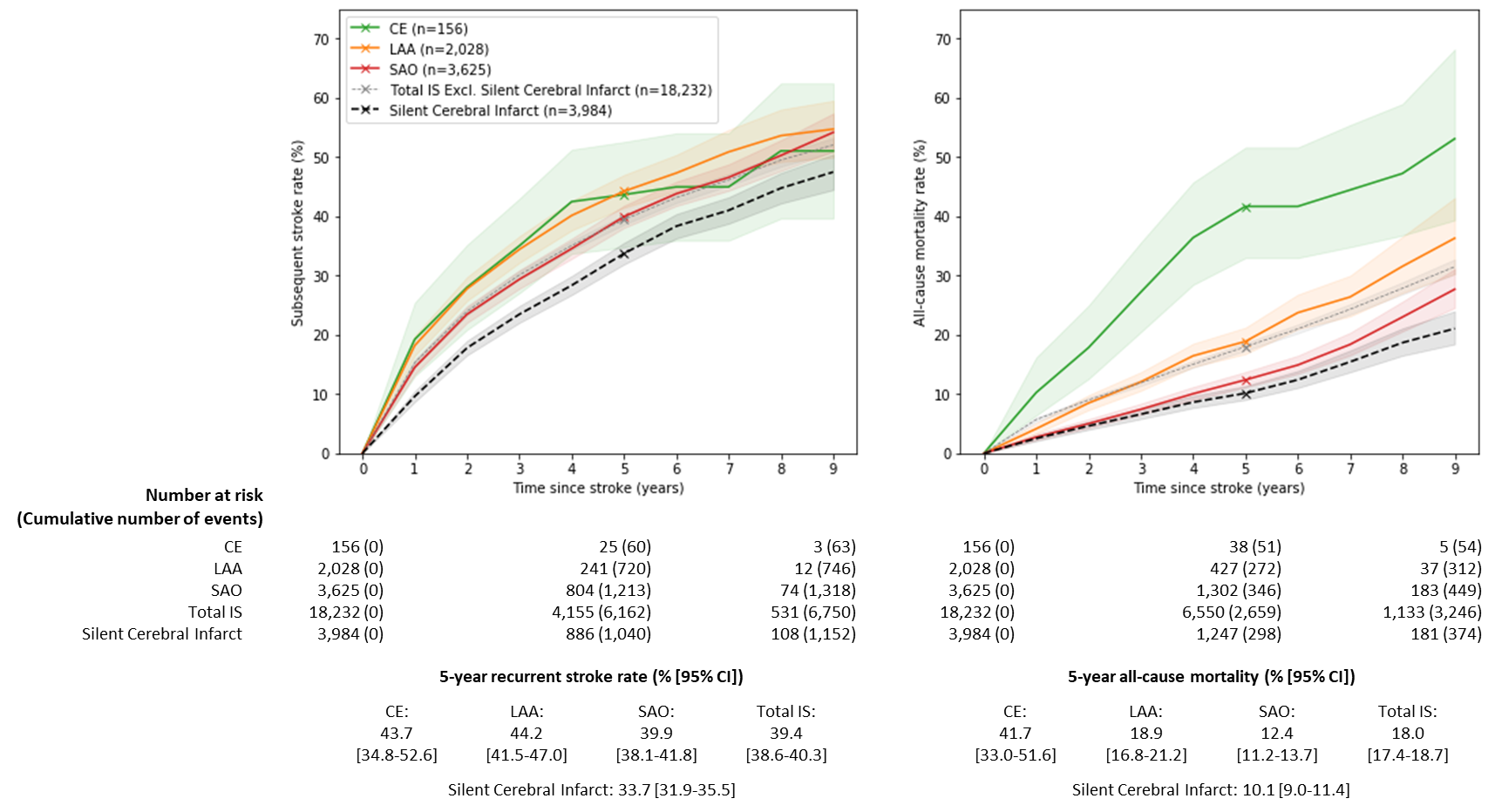
**

# **SFigure 5.** Estimated cumulative subsequent stroke and all-cause mortality rates after first ischaemic stroke (IS) of (i) different subtypes (excluding silent cerebral infarcts) and (ii) silent cerebral infarcts.

CE: Cardioaortic embolism. LAA: Large artery atherosclerosis. SAO: Small artery occlusion.

**
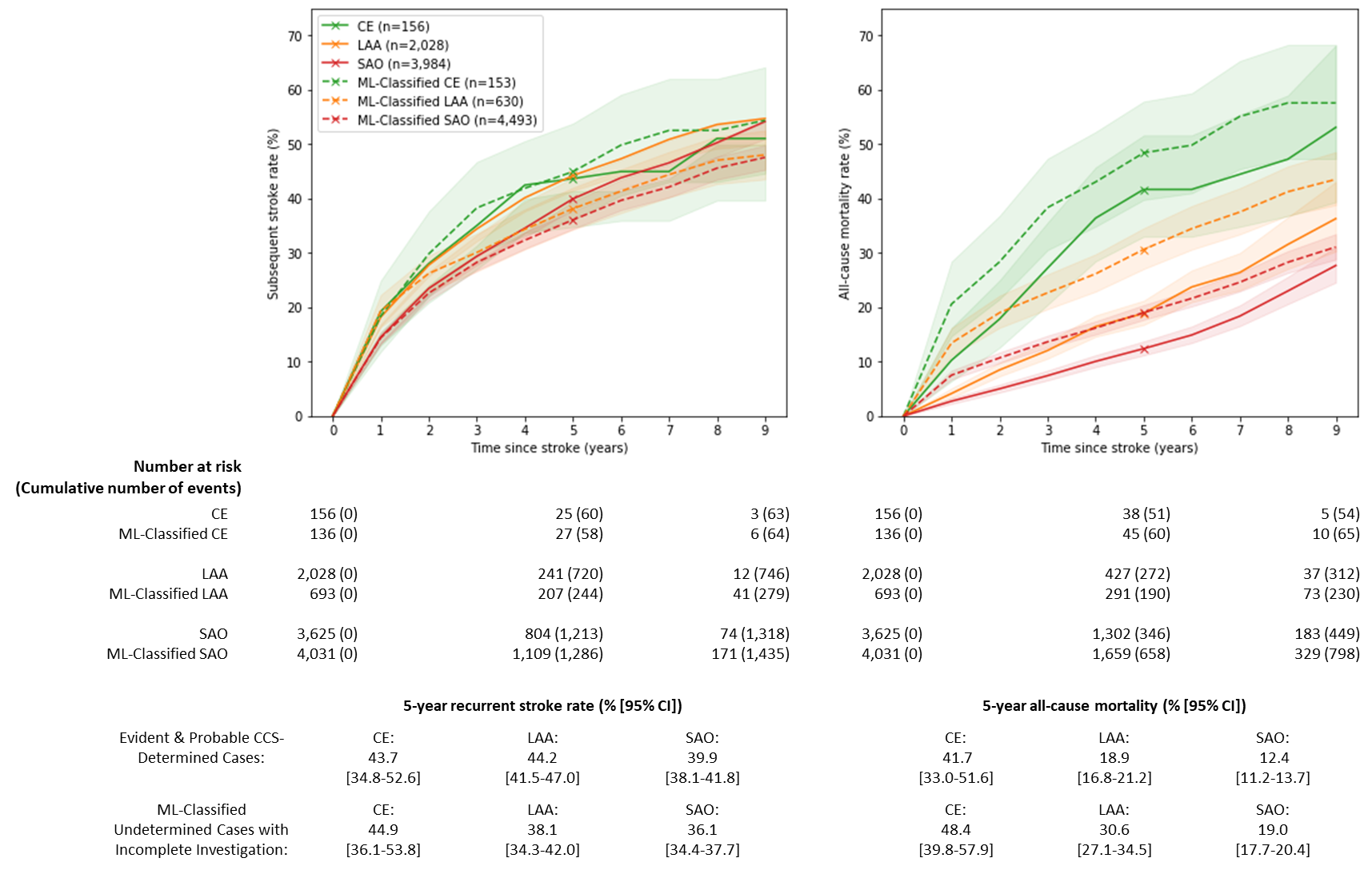
**

# **SFigure 6.** Subsequent stroke and all-cause mortality rates of different ischaemic stroke subtypes for (i) cases with evident and probable determined aetiology and (ii) machine learning (ML)-classified strokes with undetermined aetiology due to incomplete investigation (excluding silent cerebral infarcts).

CE: Cardioaortic embolism. LAA: Large artery atherosclerosis. SAO: Small artery occlusion.

# **STable 1.** List of all baseline CKB variables considered in the present study.

| **Name** | **Definition/Corresponding CKB Survey Question** |
| --- | --- |
| ***Established Risk Factor Inputs Included in 2017 Framingham Stroke Risk Profile*** | |
| Sex | Male or Female |
| Age (10 years) | Age in decades |
| Current Smoking | Do you currently smoke? (0: no, 1: yes) |
| Coronary heart disease (CHD) | Has a doctor EVER told you that you had coronary heart disease? (0: no, 1:  yes) |
| Age 65+ | Age ≥ 65 years? (0: no, 1: yes) |
| DM, if Age <65 | IF age < 65 years, do you have diabetes? (0: no, 1: yes) |
| DM, if Age 65+ | IF age < 65 years, do you have diabetes? (0: no, 1: yes) |
| HTN Rx | Do you use blood pressure drugs? (0: no, 1: yes) |
| SBP per 10 mmHg, if no HRx | IF not using blood pressure drugs, what is your systolic blood pressure (in  mmHg) / 10 |
| SBP per 10 mmHg, if HRx | IF using blood pressure drugs, what is your systolic blood pressure (in mmHg) / 10 |
| ***Geographic Variables*** | |
| region | CKB survey site region (Gansu, Haikou, Harbin, Henan, Hunan, Liuzhou, Qingdao, Sichuan, Suzhou, or Zhejiang) |
| region_is_urban | Is the region urban? (0: no, 1: yes) |
| ***Physical Measurements*** | |
| sbp_mean | Systolic blood pressure in mmHg (mean of two measurements) |
| dbp_mean | Diastolic BP in mmHg (mean of two measurements) |
| heart_rate_mean_10s | Heart rate (beats per minute/10) |
| has_diabetes | Participant has history of diabetes (reported OR random blood glucose ≥ 11.1  mmol/L OR fasting blood glucose ≥ 7.0 mmol/L) (0: no, 1: yes) |
| standing_height_cm | Standing height in cm (without shoes) |
| sitting_height_cm | Sitting height in cm |
| waist_cm | Waist measurement in cm |
| waist_hip_ratio_percent | Waist to hip ratio * 100 |
| weight_kg | Weight in kg (without shoes, but in light clothing) |
| bmi_calc | BMI calculated from measured height and weight (in kg/m^2^) |
| fat_percent | Body fat percentage |
| ***Medical History*** | |
| hypertension_diag | Has a doctor EVER told you that you had hypertension? (0: no, 1: yes) |
| has_copd | Has a doctor EVER told you that you had COPD? (0: no, 1: yes) |
| rheum_heart_dis_diag | Has a doctor EVER told you that you had rheumatic heart disease? (0: no, 1:  yes) |
| tb_diag | Has a doctor EVER told you that you had TB? (0: no, 1: yes) |
| cirrhosis_hep_diag | Has a doctor EVER told you that you had cirrhosis/chronic hepatitis? (0: no, 1: yes) |
| peptic_ulcer_diag | Has a doctor EVER told you that you had a peptic ulcer? (0: no, 1: yes) |
| gall_diag | Has a doctor EVER told you that you had a gallstone/gallbladder dis.? (0: no, 1: yes) |
| asthma_diag | Has a doctor EVER told you that you had asthma? (0: no, 1: yes) |
| kidney_dis_diag | Has a doctor EVER told you that you had kidney disease? (0: no, 1: yes) |
| fracture_diag | Has a doctor EVER told you that you had a fracture? (0: no, 1: yes) |
| rheum_arthritis_diag | Has a doctor EVER told you that you had rheumatoid arthritis? (0: no, 1: yes) |
| neurasthenia_diag | Has a doctor EVER told you that you had neurasthenia? (0: no, 1: yes) |
| head_injury_diag | Has a doctor EVER told you that you had a head injury? (0: no, 1: yes) |
| cancer_diag | Has a doctor EVER told you that you had cancer? (0: no, 1: yes) |
| blood_transfusions | How many blood transfusions have you received? (if none, put 0) |
| emph_bronc_diag | Has a doctor EVER told you that you had emphysema/bronchitis? (0: no, 1:  yes) |
| psych_disorder_diag | Has a doctor EVER told you that you have a psychiatric disorder? (0: no, 1:  yes) |
| ***Family Medical History*** | |
| children | How many children do you have? |
| siblings | How many siblings do you have? |
| mother_still_alive | Is your mother still alive? (0: no, 1: yes) |
| father_still_alive | Is your father still alive? (0: no, 1: yes) |
| mother_stroke | Did your mother ever have a stroke? (0: no, 1: yes) |
| mother_heart_attack | Did your mother ever have a heart attack? (0: no, 1: yes) |
| mother_diabetes | Did your mother ever have diabetes? (0: no, 1: yes) |
| mother_cancer | Did your mother ever have cancer? (0: no, 1: yes) |
| father_stroke | Did your father ever have a stroke? (0: no, 1: yes) |
| father_heart_attack | Did your father ever have a heart attack? (0: no, 1: yes) |
| father_diabetes | Did your father ever have diabetes? (0: no, 1: yes) |
| father_cancer | Did your father ever have cancer? (0: no, 1: yes) |
| siblings_stroke | Did your siblings (incl. half siblings) ever have a stroke? (0: if no, else record the number with disease) |
| siblings_heart_attack | Did your siblings (incl. half siblings) ever have a heart attack? (0: if no, else record the number with disease) |
| siblings_diabetes | Did your siblings (incl. half siblings) ever have diabetes? (0: if no, else record the number with disease) |
| siblings_cancer | Did your siblings (incl. half siblings) ever have cancer? (0: if no, else record  the number with disease) |
| children_stroke | Did your children ever have a stroke? (0: if no, else record the number with  disease) |
| children_heart_attack | Did your children ever have a heart attack? (0: if no, else record the number with disease) |
| children_diabetes | Did your children ever have diabetes? (0: if no, else record the number with  disease) |
| children_cancer | Did your children ever have cancer? (0: if no, else record the number with  disease) |
| missing_mother_history | Missing reported history of stroke, heart attack, diabetes, and/or cancer in  mother |
| missing_father_history | Missing reported history of stroke, heart attack, diabetes, and/or cancer in  father |
| missing_siblings_history | Missing reported history of stroke, heart attack, diabetes, and/or cancer in  siblings |
| ***Lifestyle Factors*** | |
| met | Total daily physical activity (Metabolic Equivalent of Task [MET hours/day]) |
| met_hours | Total daily hours spent on physical activity |
| smoking_category_1 | Does the following characterise your smoking behaviour? – Never smoker (0: no, 1: yes) |
| smoking_category_2 | Does the following characterise your smoking behaviour? – Occasional smoker (0: no, 1: yes) |
| smoking_category_3 | Does the following characterise your smoking behaviour? – Ex regular smoker (0: no, 1: yes) |
| smoking_category_4 | Does the following characterise your smoking behaviour? – Regular smoker (0: no, 1: yes) |
| smoking_now_1 | Does the following characterise your smoking behaviour? – Only occasionally (0: no; 1: yes) |
| smoking_now_2 | Does the following characterise your smoking behaviour? – Yes, on most days (0: no; 1: yes) |
| smoking_now_3 | Does the following characterise your smoking behaviour? – Yes, daily or almost every day (0: no; 1: yes) |
| years_since_quitting_smoking | How many years since quitting smoking? (0 if you currently smoke or never smoked; decimal values allowed) |
| alcohol_category_1 | Does the following characterise your alcohol intake? – Never regular (0: no; 1: yes) |
| alcohol_category_2 | Does the following characterise your alcohol intake? – Ex-regular (0: no; 1:  yes) |
| alcohol_category_3 | Does the following characterise your alcohol intake? – Occasional (0: no; 1: yes) |
| alcohol_category_4 | Does the following characterise your alcohol intake? – Monthly (0: no; 1: yes) |
| alcohol_category_5 | Does the following characterise your alcohol intake? – Reduced intake (0: no; 1: yes) |
| alcohol_category_6 | Does the following characterise your alcohol intake? – Weekly (0: no; 1: yes) |
| diet_freq_rice_0 | During the past 12 months, about how often did you eat rice? – Daily (0: no,  1: yes) |
| diet_freq_rice_1 | During the past 12 months, about how often did you eat rice? – 4-6 days per  week (0: no, 1: yes) |
| diet_freq_rice_2 | During the past 12 months, about how often did you eat rice? – 1-3 days per  week (0: no, 1: yes) |
| diet_freq_rice_3 | During the past 12 months, about how often did you eat rice? – Monthly (0:  no, 1: yes) |
| diet_freq_rice_4 | During the past 12 months, about how often did you eat rice? – Never/rarely  (0: no, 1: yes) |
| diet_freq_wheat_0 | During the past 12 months, about how often did you eat wheat? – Daily (0: no,  1: yes) |
| diet_freq_wheat_1 | During the past 12 months, about how often did you eat wheat? – 4-6 days per  week (0: no, 1: yes) |
| diet_freq_wheat_2 | During the past 12 months, about how often did you eat wheat? – 1-3 days per  week (0: no, 1: yes) |
| diet_freq_wheat_3 | During the past 12 months, about how often did you eat wheat? – Monthly (0:  no, 1: yes) |
| diet_freq_wheat_4 | During the past 12 months, about how often did you eat wheat? – Never/rarely  (0: no, 1: yes) |
| diet_freq_other_staple_0 | During the past 12 months, about how often did you eat staple foods other  than rice and wheat (corn, millet etc.)? – Daily (0: no, 1: yes) |
| diet_freq_other_staple_1 | During the past 12 months, about how often did you eat staple foods other  than rice and wheat (corn, millet etc.)? – 4-6 days per week (0: no, 1: yes) |
| diet_freq_other_staple_2 | During the past 12 months, about how often did you eat staple foods other  than rice and wheat (corn, millet etc.)? – 1-3 days per week (0: no, 1: yes) |
| diet_freq_other_staple_3 | During the past 12 months, about how often did you eat staple foods other  than rice and wheat (corn, millet etc.)? – Monthly (0: no, 1: yes) |
| diet_freq_other_staple_4 | During the past 12 months, about how often did you eat staple foods other  than rice and wheat (corn, millet etc.)? – Never/rarely (0: no, 1: yes) |
| bowel_movement_freq_0 | About how often do you have bowel movements each week? – More than once on most days (0: no, 1:yes) |
| bowel_movement_freq_1 | About how often do you have bowel movements each week? – About daily (0: no, 1:yes) |
| bowel_movement_freq_2 | About how often do you have bowel movements each week? – Once every 2-3 days (0: no, 1:yes) |
| bowel_movement_freq_3 | About how often do you have bowel movements each week? – Less than 3  times a week (0: no, 1:yes) |
| gum_bleed_freq_0 | How often do your gums bleed when you brush your teeth? – Occasionally,  rarely or never (0: no, 1:yes) |
| gum_bleed_freq_1 | How often do your gums bleed when you brush your teeth? – Sometimes (0:  no, 1:yes) |
| gum_bleed_freq_2 | How often do your gums bleed when you brush your teeth? – Always (0: no,  1:yes) |
| gum_bleed_freq_3 | How often do your gums bleed when you brush your teeth? – Brush teeth  rarely or never (0: no, 1:yes) |
| ***Socioeconomic Demographic Factors*** | |
| household_size | How many people live together in your household? |
| has_health_cover | Do you have health care coverage? (0: no, 1: yes) |
| highest_education_0 | What is the highest level of school education you ever received? – No formal school (0: no, 1: yes) |
| highest_education_1 | What is the highest level of school education you ever received? – Primary school (0: no, 1: yes) |
| highest_education_2 | What is the highest level of school education you ever received? – Middle  school (0: no, 1: yes) |
| highest_education_3 | What is the highest level of school education you ever received? – High school (0: no, 1: yes) |
| highest_education_4 | What is the highest level of school education you ever received? – Technical school / college (0: no, 1: yes) |
| highest_education_5 | What is the highest level of school education you ever received? – Technical school / college (0: no, 1: yes) |
| occupation_0 | What is your current occupation? – Agriculture and related (0: no, 1: yes) |
| occupation_1 | What is your current occupation? – Factory worker (0: no, 1: yes) |
| occupation_2 | What is your current occupation? – Administrator / manager (0: no, 1: yes) |
| occupation_3 | What is your current occupation? – Professional / technical (0: no, 1: yes) |
| occupation_4 | What is your current occupation? – Sales and service (0: no, 1: yes) |
| occupation_5 | What is your current occupation? – Retired (0: no, 1: yes) |
| occupation_6 | What is your current occupation? – House wife / husband (0: no, 1: yes) |
| occupation_7 | What is your current occupation? – Self-employed (0: no, 1: yes) |
| occupation_8 | What is your current occupation? – Unemployed (0: no, 1: yes) |
| occupation_9 | What is your current occupation? – Other or not stated (0: no, 1: yes) |
| household_income_0 | What is the total income last year in your household? – <2,500 yuan (0: no, 1: yes) |
| household_income_1 | What is the total income last year in your household? – 2,500-4,999 yuan (0: no, 1: yes) |
| household_income_2 | What is the total income last year in your household? – 5,000-9,999 yuan (0: no, 1: yes) |
| household_income_3 | What is the total income last year in your household? – 10,000-19,999 yuan (0: no, 1: yes) |
| household_income_4 | What is the total income last year in your household? – 20,000-34,999 yuan (0: no, 1: yes) |
| household_income_5 | What is the total income last year in your household? – ≥35,000 yuan (0: no, 1: yes) |
| ***Self-Assessed Health Status*** | |
| self_rated_health_0 | How would you rate your current general health status? – Excellent (0: no, 1: yes) |
| self_rated_health_1 | How would you rate your current general health status? – Good (0: no, 1: yes) |
| self_rated_health_2 | How would you rate your current general health status? – Fair (0: no, 1: yes) |
| self_rated_health_3 | How would you rate your current general health status? – Poor (0: no, 1: yes) |
| comparative_health_0 | How would rate your current general health status compared to someone of  your own age? – Better (0: no, 1: yes) |
| comparative_health_1 | How would rate your current general health status compared to someone of  your own age? – About the same (0: no, 1: yes) |
| comparative_health_2 | How would rate your current general health status compared to someone of  your own age? – Worse (0: no, 1: yes) |
| comparative_health_3 | How would rate your current general health status compared to someone of  your own age? – Don’t know (0: no, 1: yes) |

# STable 2: Copy of adjudication form used by Chinese Clinicians to classify stroke types

**Overview of case adjudication**

Patient ID: 120345678

PVD disease type

Discharge diagnosis

Clinical presentation

Physical presentation

Clinical Investigation and Main findings of tests.

CT

MRI

Doctor’s adjudication results:

Doctor’s level of confidence

Doctor’s comments

Date:

**Duration of prior medical history**

| Unknown | <1 month | <1 year | 1-5 years | > 5 years | > 10 years |
| --- | --- | --- | --- | --- | --- |

**Duration of symptoms**

| <24 hours | > 24 hours < 7 days | > 7 days < 28 days | > 28 days | >Other specify) |
| --- | --- | --- | --- | --- |

**Test report**

Formal test report

Results described in the note

Results not reported anywhere in the note

**Testing hospital**

In this hospital  Yes  No

**Confidence level of adjudicator**

Definite

Probably

Possible

Unlikely

**Stroke-death**

Died of stroke

Died of other non-stroke neurological deficits

Died of other causes, please specify

**Stroke List**

**Reported classification**

Stroke (NOS)

Ischaemic stroke (IS)

Lacunar infarct (LACI)

Intracerebral haemorrhage (ICH)

Subarachnoid haemorrhage (SAH)

**Hospital classification**

Stroke (NOS)

Non-LACI Ischaemic stroke (IS)

Intracerebral haemorrhage (ICH)

Subarachnoid haemorrhage (SAH)

Old stroke (>1 month)

Other neurological deficits, if yes, specify ______________

**Adjudication classification (confirmed)**

Stroke (NOS)

Non-LACI Ischaemic stroke (IS)

Lacunar infarct (LACI) (with neurological deficits)

ICH transformation after LACI (LACI + ICH)

ICH transformation after IS (IS + ICH)

ICH with extension to SAH (ICH + SAH)

Subarachnoid haemorrhage (SAH)

SAH with ischaemic changes (SAH + IS)

Old stroke (>1 month (go to Old-1.1)

Insufficient evidence

**Adjudication classification (refuted)**

TIA

Imaging detected infarction (Silent LACI without neurological deficits)

Other neurological disorders, if yes, specify

Other non-neurological disorders, if yes, specify

Insufficient evidence

**Old stroke cases**

Old Stroke (Old NOS)

Old Ischaemic stroke (Old IS)

Old Lacunar infarct (Old LACI)

Old Intracerebral haemorrhage (Old ICH)

Old Subarachnoid haemorrhage (Old SAH)

**Differential diagnoses**

**Yes No/unknown**

Brain tumour (including metastatic tumour)

Head or neck injury

Epilepsy

Ear diseases

Other neurological abnormalities

If yes, specify _____________

**Complications and co-morbidities**

**Yes No/Unknown**

Dementia

Hypertension

Heart disease

Cancer

COPD

Diabetes Mellitus

TCM diagnoses

Other, specify _______

**Known risk factors**

**Yes No/Unknown**

Hypertension

Diabetes mellitus (DM)

Transient ischaemic attack (TIA)

Atrial Fibrillation (AF)

Intermittent claudication

Systemic embolism

Carotid bruit

Diminished pulse

High blood lipids

Use of anticoagulation drugs within 6 months before admission

Use of anti-platelet medicines within 6 months before admission

CEA before admission

Stenting (incl. intra-and extra-cranial)

**Clinical presentation (presenting symptoms)**

**Yes No/Unknown**

Sudden face weakness

Weakness in one arm or leg

Language or speech difficulties

Sudden decrease in the levels of consciousness

Other, specify______________

**Physical examination**

**Yes No/Unknown**

Coma or consciousness problem

Neck stiffness

Unilateral weakness (and/or sensory deficit) affecting face

Unilateral weakness (and/or sensory deficit) affecting arm or hand

Unilateral weakness (and/or sensory deficit) affecting leg or foot

Dysphasia

Homnymous hemianopia

Monocular or binocular visual loss

Diplopia

Visual field deficits

Visuospatial disorder

(e.g. sensory or visual inattention, unable to copy pictures)

Brainstem or cerebellar signs (e.g. nystagmus or ataxia)

Pattern of neurological deficit unclear

Atrial Fibrillation

Evidence of systemic embolization

Pure motor

Pure sensory

Mixed sensorimotor

Ataxic hemiparesis

Dysarthria

Clumsy hand

Other neurological deficit, if yes, specify _______________

Was above examination done by neurologist;  Yes;  No;

**Clinical Investigations**

**Yes No/Unknown**

CT

MRI

Cerebral artery tests (Extra-cranial)

Cerebral artery tests (Intra-cranial)

ECG

Holter

Echocardiogram

Specialized tests .

CSF

Blood tests for [hypercoagulable state](http://www.iciba.com/hypercoagulative_state)s

Any other tests, specify_____________________

**MF-CT/MRI-brain**

Normal

Haemorrhagic lesion

Ischemic changes (>1.5cm)

Irregular shape and undefined size (eg, lacunae) (≤1.5cm)

ICH transformation after initial IS

Mainly haemorrhagic lesion with ischemic changes

Soften lesion (old lesion)

Blood in the subarachnoid space

Other (including multiple lesions which are difficult to categorize), specify__

**Laterality of lesions**

Left

Right

Both

**Location of brain lesions**

**Yes No/Unknown**

Frontal lobe

Temporal lobe

Parietal lobe

Occipital lobe

Deep regions

*(Any of internal Capsule, Thalamus, Claustrum, Basal ganglia, Corona radiate, Corpus callosum)*

Cerebellum

Brain stem (midbrain, Pons, medulla)

Limbic system
(Thalamus, Hypothalamus, Amygdala, Hippocampus),

Location not noted

Other location, specify ___

**No. of lesions**

| 1 | 2-5 | >5 | Not noted |
| --- | --- | --- | --- |

**Location of artery lesions**

**Yes No/Unknown *Extra-cranial***

***Common carotid artery

Carotid bifurcation

***Internal Carotid artery

***External Carotid artery

Vertebral artery

***Subclavian artery

***Intracranial

***Middle cerebral artery

***Anterior cerebral artery

***Posterior cerebral artery

***Internal carotid artery

Vertebral artery

***Basilar Artery

**Degree of stenosis**

No narrowing or narrowing with unknown degree

Not smooth, or <30%

30-49%

50-69%

70-99%

100%

**Method for calculating stenosis**

Unknown

North American Symptomatic Carotid Endarterectomy Trial (NASCET)

European Carotid Surgery Trial (ECST)

Common Carotid (CC)

Carotid Stenosis Index (CSI)

Other

**Carotid artery ultrasound features of carotid plaques**

Hyperechoic plaque

Hypoechoic plaque

Mixed echoic plaque

Unknown

IMT value ______mm.

**Imaging methods**

**Yes No/Unknown**

T1 &T2

Diffusion Weighted Imaging (DWI)

Perfusion Weighted Imaging (PWI)

T2

Susceptibility Weighted Imaging (SWI)

Other, specify______

**Intra-cranial**

Digital Subtraction Angiography (DSA)

Computed Tomography Angiography (CTA)

Magnetic Resonance Angiography (MRA)

Contrast-Enhanced MRA (CEMRA)

Trans-Cranial Doppler (TCD)

Other, specify______

**Extra-cranial**

Digital Subtraction Angiography (DSA)

Computed Tomography Angiography (CTA)

Contrast-Enhanced MRA (CEMRA)

Intravascular Ultrasound (IVUS)

Other, specify______

**Cardiac sources**

**Yes No/Unknown**

Atrial fibrillation (Demonstrated at any time during hospitalisation)

Acute MI

Sick sinus syndrome

Atrial flutter (Demonstrated at any time during hospitalisation)

Other, specify______

**Other cardiac sources**

**Yes No/Unknown**

Mechanical Prosthetic Valve

Atrial Fibrillation

Sick Sinus Syndrome

Myocardial infarction (MI) within 4 Weeks

Dilated Cardiomyopathy

Atrial Myxoma

Infective Endocarditis

Akinetic Left Ventricular Segment

Left Ventricular Thrombus

MI > 4 weeks ago but < 6 months ago

Congestive Heart Failure

Left Ventricular Aneurysm

Atrial Flutter

Bioprosthetic Heart Valve

Mitral Stenosis without Atrial Fibrillation

Mitral Valve Prolapsed

Mitral Annulus Calcification

Atrial Septal Defect

Patent Foramen Ovale

Interatrial Septal Aneurysm

Nonbacterial Endocarditis

Other, specify______

**Echocardiography**

**Yes No**

Transthoracic echocardiogram (TTE)

Transesophageal echocardiogram (TEE)

Stress echocardiography

3D echocardiography (color)

Contrast echocardiography

Other, specify______

**Treatment**

**Stroke treatment No/Unknown <24HR Admission Discharge**

**Any reperfusion therapy**

r-tPA I.V

r-tPA I.A

Urokinase I.V

Urokinase I.A

mechanical embolectomy

Any antiplatelet

Aspirin plus clopidogrel

Aspirin alone

Clopidogrel alone

Any anticoagulant

LMWH

UFH

Warfarin

Statin

Non-Statin

**Revascularisation therapy**

Stenting

Coiling

**Surgery**

CEA

Decompressive craniectomy

Surgical *removal* *of* intracranial *hematoma*

Minimally invasive clot evacuation

Aneurysm clipping surgery.

**Antihypertension and other medication**

ACE inhibitors

Calcium channel blockers

ACE receptor blockers

Diuretics

*β*-blockers

Defibrase

Any Traditonal Chinese Medicines

Other, specify (free text)_____________________

# **STable 3:** Key variables collected from medical records for classification of IS subtypes using CCS.

| **Name** | **Value Definitions** |
| --- | --- |
| Imaging Findings | - No brain imaging - Normal imaging - “Ischaemic (non-lacunar)”: >1.5cm infarct - “Ischaemic (lacunar)”: ≤1.5cm infarct. Irregular shape and undefined size (e.g., lacunae) - Indeterminate imaging |
| intra_extra_test_done | - No vascular imaging - Intracranial (IC) vascular imaging only - Extracranial (EC) vascular imaging only - Both IC and EC vascular imaging |
| intra_extra_is_abnormal | - No stenosis in either IC or EC artery - Any stenosis in IC artery only - Any stenosis in EC artery only - Any stenosis in both IC and EC artery |
| intra_extra_loc_art_dgre_stsis | - No stenosis ≥50% in either IC or EC artery - Any stenosis ≥50% in IC artery only - Any stenosis ≥50% in EC artery only - Any stenosis ≥50% in both IC and EC artery |
| ultrasound_plaq_featre | - NULL: No plaque - 0: If plaque, stable (hyperechoic) – EC artery only - 1: If plaque, unstable (hypoechoic or mixed echoic) – EC artery only |
| echo_ecg_test_done | - No cardiac test done - Electrocardiogram (ECG) only - Echocardiogram (Echo) only - Both ECG and Echo |
| ce_risk | - 0: No cardioembolic source - 1: Low- or uncertain risk cardioembolic source (e.g., mitral annular calcification, patent foramen ovale) - 2: High-risk cardioembolic source (e.g., atrial fibrillation, mechanical prosthetic valve) |
| systemic_embolization | - 0: No evidence of systemic embolism - 1: Evidence of systemic embolism |
| lacunar_syndrome | - 0: No clinical lacunar syndrome - 1: Presence of clinical lacunar syndrome (e.g., pure motor, pure sensory, ataxic hemiparesis, clumsy hand) |

# STable 4: Breakdown of clinical investigations for aetiologically-classified ischaemic stroke (IS) subtypes of evident or probable confidence level and ML-classified IS subtypes.

|  | **Aetiologically-Classified Evident and Probable IS Subtypes** | | |  | **ML-Classified IS Subtypes** | | |
| --- | --- | --- | --- | --- | --- | --- | --- |
|  | **CE**  No. of Events (%) | **LAA**  No. of Events (%) | **SAO**  No. of Events (%) |  | **CE**  No. of Events (%) | **LAA**  No. of Events (%) | **SAO**  No. of Events (%) |
| **Brain Imaging** |  |  |  |  |  |  |  |
| CT and MRI | 91 (57) | 831 (35) | 1,508 (31) |  | 18 (12) | 51 (8) | 380 (8) |
| CT Only | 56 (35) | 1145 (48) | 2,454 (50) |  | 116 (76) | 423 (67) | 2,265 (50) |
| MRI Only | 13 (8) | 363 (15) | 926 (19) |  | 18 (12) | 20 (3) | 450 (10) |
| Neither | 0 (0) | 56 (2) | 0 (0) |  | 1 (1) | 136 (22) | 1,398 (31) |
| **Cardiac Tests** |  |  |  |  |  |  |  |
| Echo and ECG | 121 (7%) | 1,254 (52) | 2,025 (41) |  | 81 (53) | 39 (6) | 479 (11) |
| Echo Only | 5 (3) | 56 (2) | 119 (2) |  | 1 (1) | 1 (0) | 36 (1) |
| ECG Only | 34 (21) | 1,035 (43) | 2,676 (55) |  | 71 (46) | 465 (74) | 2,628 (58) |
| Neither | 0 (0) | 50 (2) | 68 (1) |  | 0 (0) | 125 (20) | 1,350 (30) |
| **Vascular Imaging** |  |  |  |  |  |  |  |
| IC and EC | 62 (39) | 1,222 (51) | 1,553 (32) |  | 0 (0) | 12 (2) | 122 (3) |
| IC Only | 45 (28) | 46 (2) | 1,639 (34) |  | 0 (0) | 20 (3) | 225 (5) |
| EC Only | 50 (31) | 1,127 (47) | 1,428 (29) |  | 0 (0) | 21 (3) | 133 (3) |
| Neither | 3 (2) | 0 (0) | 268 (5) |  | 153 (100) | 577 (92) | 4,013 (89) |

CE: Cardioaortic embolism. LAA: Large artery atherosclerosis. SAO: Small artery occlusion. CT: Computerised tomography. MRI: Magnetic resonance imaging. Echo: Echocardiography. ECG: Electrocardiogram. IC: Intracranial vascular imaging. EC: Extracranial vascular imaging.

# **STable 5:** Breakdown of second strokes by pathological types following a first stroke of different ischaemic stroke (IS) subtypes.

|  | **First IS Subtype** | | |
| --- | --- | --- | --- |
|  | **CE**  Number of Events (%) | **LAA**  Number of Events (%) | **SAO**  Number of Events (%) |
| **Second Stroke Type** |  |  |  |
| IS (I63) | 60 (94) | 766 (91) | 1,506 (91) |
| ICH (I61) | 4 (6) | 51 (6) | 85 (5) |
| SAH (I60) | 0 (0) | 0 (0) | 5 (0) |
| Unspecified (I64) | 0 (0) | 26 (3) | 68 (4) |

CE: Cardioaortic embolism. LAA: Large artery atherosclerosis. SAO: Small artery occlusion. ICH: Intracerebral haemorrhage. SAH: Subarachnoid haemorrhage.

# **STable 6:** Top risk factors for classifying ischaemic stroke (IS) subtypes.

| **CE** | |  | **LAA** | |  | **SAO** | |
| --- | --- | --- | --- | --- | --- | --- | --- |
| Risk Factor | OR  [95% CI] |  | Risk Factor | OR  [95% CI] |  | Risk Factor | OR  [95% CI] |
| ce_risk | 84,607  [2.1*10^4^­–2.2*10^6^] |  | Hunan | 2.81  [2.21-3.94] |  | ce_risk | 0.13  [0.09-0.17] |
| Age (10 years) | 0.20  [0.05 – 0.84] |  | Harbin | 0.55  [0.43-0.71] |  | Hunan | 0.34  [0.25-0.44] |
| BMI | 3.12  [1.98-6.11] |  | Female | 0.59  [0.46-0.73] |  | Harbin | 1.92  [1.44-2.45] |
| Fat percent | 0.77  [0.68-0.91] |  | Suzhou | 1.64  [1.17-2.30] |  | Suzhou | 0.57  [0.41-0.85] |
|  |  |  | Henan | 0.68  [0.47-0.97] |  | Female | 1.74  [1.41-2.31] |
|  |  |  | Haikou | 1.45  [1.08-1.98] |  | Henan | 1.48  [1.03-2.04] |
|  |  |  | Liuzhou | 0.74  [0.54-0.99] |  | Haikou | 0.69  [0.50-0.92] |
|  |  |  | Health cover | 0.79  [0.63-0.97] |  | Liuzhou | 1.35  [1.00-1.82] |
|  |  |  | Father stroke | 0.86  [0.69-0.97] |  | Health cover | 1.29  [1.08-1.60] |
|  |  |  | SBP_noHRX | 1.10  [1.03-1.26] |  | Father stroke | 1.17  [1.03-1.45] |

CE: Cardioaortic embolism. LAA: Large artery atherosclerosis. SAO: Small artery occlusion.

Risk factors ranked by magnitude of odds ratio (OR) and included if 95% CI indicated statistical significance. Here, the reported ORs do not represent the increased or decreased likelihood of a particular IS subtype occurring given the presence of a risk factor. Instead, the ORs represent the increased or decreased likelihood that an existing stroke belongs to a particular IS subtype given the presence of the risk factor. Red cells indicate increased likelihood of corresponding IS subtype. Green cells indicate decreased likelihood of corresponding IS subtype.

# **STable 7:** Breakdown of primary ischaemic stroke (IS) cases with separation of silent and non-silent strokes.

| **CCS Aetiological Subtype** | **Total Primary IS Cases**  n= 22,216 | **Non-Silent Stroke**  n= 18,232 | **Silent Cerebral Infarct**  n= 3,984 |
| --- | --- | --- | --- |
| Determined Cases |  |  |  |
| LAA-evident | 888 (4%) | 862 (5%) | 26 (1%) |
| LAA-probable | 1,507 (7%) | 1,166 (6%) | 341 (9%) |
| LAA-possible | 1,953 (9%) | 1,872 (10%) | 81 (2%) |
|  |  |  |  |
| CE-evident | 157 (1%) | 153 (1%) | 4 (0%) |
| CE-probable | 3 (0%) | 3 (0%) | 0 (0%) |
| CE-possible | 235 (1%) | 228 (1%) | 7 (0%) |
|  |  |  |  |
| SAO-evident | 4,620 (21%) | 3,366 (18%) | 1,254 (31%) |
| SAO-probable | 268 (1%) | 259 (1%) | 9 (0%) |
| SAO-possible | 5,900 (27%) | 4,284 (23%) | 1,616 (41%) |
| Undetermined Cases |  |  |  |
| >1 identified likely cause: | 1,008 (5%) | 864 (5%) | 144 (4%) |
| No identified likely cause: | 5,677 (26%) | 5,175 (28%) | 502 (13%) |

CE: Cardioaortic embolism. LAA: Large artery atherosclerosis. SAO: Small artery occlusion.

# **STable 8:** Top risk factors for classifying ischaemic stroke (IS) subtypes (excluding silent cerebral infarcts).

| **CE** | |  | **LAA** | |  | **SAO** | |
| --- | --- | --- | --- | --- | --- | --- | --- |
| Risk Factor | OR  [95% CI] |  | Risk Factor | OR  [95% CI] |  | Risk Factor | OR  [95% CI] |
| ce_risk | 131  [106-168] |  | Hunan | 2.98  [2.21-3.94] |  | ce_risk | 0.15  [0.11-0.19] |
| Children | 0.68  [0.51 – 0.88] |  | Female | 0.57  [0.46-0.74] |  | Hunan | 0.32  [0.24-0.44] |
|  |  |  | Harbin | 0.58  [0.46-0.73] |  | Female | 1.84  [1.39-2.28] |
|  |  |  | Suzhou | 1.57  [1.16-2.52] |  | Harbin | 1.74  [1.38-2.23] |
|  |  |  | Haikou | 1.50  [1.06-1.98] |  | Suzhou | 0.60  [0.41-0.82] |
|  |  |  | Eats wheat monthly | 0.81  [0.69-1.00] |  | Haikou | 0.68  [0.52-0.92] |
|  |  |  | Father stroke | 0.83  [0.68-0.99] |  | Father stroke | 1.22  [1.01-1.44] |
|  |  |  | Household size | 1.07  [1.02-1.10] |  | Eats wheat monthly | 1.20  [1.01-1.45] |
|  |  |  | SBP_noHRX | 1.06  [1.01-1.14] |  | Household size | 0.94  [0.90-0.98] |
|  |  |  |  |  |  | Physical Activity (met hours) | 1.01  [1.00-1.03] |

CE: Cardioaortic embolism. LAA: Large artery atherosclerosis. SAO: Small artery occlusion.

Risk factors ranked by magnitude of odds ratio (OR) and included if 95% CI indicated statistical significance. Here, the reported ORs do not represent the increased or decreased likelihood of a particular IS subtype occurring given the presence of a risk factor. Instead, the ORs represent the increased or decreased likelihood that an existing stroke belongs to a particular IS subtype given the presence of the risk factor. Red cells indicate increased likelihood of corresponding IS subtype. Green cells indicate decreased likelihood of corresponding IS subtype.

# Supplementary References

1. Ay H, Furie KL, Singhal A, Smith WS, Sorensen AG, and Koroshetz WJ. An Evidence-Based Causative Classification System for Acute Ischemic Stroke. *Ann Neurol*. 2005;58:688–697.
2. Ay H, Benner T, Arsava M, Furie KL, Singhal AB, Jensen MB, et al. A Computerized Algorithm for Etiologic Classification of Ischemic Stroke: The Causative Classification of Stroke System. *Stroke*. 2007;38:2979–2984.
3. Pedregosa F, Varoquaux G, Gramfort A, et al. Scikit-learn: machine learning in Python. *JMLR*. 2011;12:2825-30.
4. Arsava EM, Helenius J, Avery R, Sorgun MH, Kim G and Pontes-Neto OM. Assessment of the Predictive Validity of Etiologic Stroke Classification. *JAMA Neurol*. 2017;74:419-426.
